# Gene expression profiling of the venomgland from the Venezuelan mapanare (*Bothrops colombiensis*) using expressed sequence tags (ESTs)

**DOI:** 10.1186/s12867-016-0059-7

**Published:** 2016-03-05

**Authors:** Montamas Suntravat, Néstor L. Uzcategui, Chairat Atphaisit, Thomas J. Helmke, Sara E. Lucena, Elda E. Sánchez, Alexis Rodríguez Acosta

**Affiliations:** National Natural Toxins Research Center, Department of Chemistry, Texas A and M University-Kingsville, Kingsville, USA; Laboratorio de Inmunoquímica y Ultraestructura, Instituto Anatómico de la Universidad Central de Venezuela, Caracas, Venezuela

**Keywords:** cDNA library, *Bothrops colombiensis*, Viperidae, Expressed sequence tags

## Abstract

**Background:**

*Bothrops colombiensis* is a highly dangerous pit viper and responsible for over 70 % of snakebites in Venezuela. Although the composition in *B. colombiensis* venom has been identified using a proteome analysis, the venom gland transcriptome is currently lacking.

**Results:**

We constructed a cDNA library from the venom gland of *B. colombiensis*, and a set of 729 high quality expressed sequence tags (ESTs) was identified. A total number of 344 ESTs (47.2 % of total ESTs) was related to toxins. The most abundant toxin transcripts were metalloproteinases (37.5 %), phospholipases A_2_s (PLA_2_, 29.7 %), and serine proteinases (11.9 %). Minor toxin transcripts were linked to waprins (5.5 %), C-type lectins (4.1 %), ATPases (2.9 %), cysteine-rich secretory proteins (CRISP, 2.3 %), snake venom vascular endothelium growth factors (svVEGF, 2.3 %), L-amino acid oxidases (2 %), and other putative toxins (1.7 %). While 160 ESTs (22 % of total ESTs) coded for translation proteins, regulatory proteins, ribosomal proteins, elongation factors, release factors, metabolic proteins, and immune response proteins. Other proteins detected in the transcriptome (87 ESTs, 11.9 % of total ESTs) were undescribed proteins with unknown functions. The remaining 138 (18.9 %) cDNAs had no match with known GenBank accessions.

**Conclusion:**

This study represents the analysis of transcript expressions and provides a physical resource of unique genes for further study of gene function and the development of novel molecules for medical applications.

**Electronic supplementary material:**

The online version of this article (doi:10.1186/s12867-016-0059-7) contains supplementary material, which is available to authorized users.

## Background

Snake venoms, mainly from Viperidae families are rich reservoirs of metalloproteinases, serine proteinases, and phospholipase A_2_ (PLA_2_) [1–6], inducing a diversity of hemostatic effects such as blood coagulation, hemorrhage, and platelet aggregation. Hemorrhage is mainly caused by snake venom zinc-dependent metalloproteinases, which digest components of the extracellular matrix (ECM) proteins resulting in bleedings [4].

*Bothrops* snakes belonging to the family Viperidae are the major cause of snakebite morbidity and mortality in Central and South America [7]. *Bothrops colombiensis* bites were responsible for over 70 % of accidents in Venezuela annually [8, 9]. Symptoms of Bothropoid envenoming include edema, pain, myonecrosis, hemorrhage, and systemic effects such as hemostatic disorders and cardiovascular shock [10]. Using a proteome analysis, many biological proteins, mainly metalloproteinases and PLA_2_, were identified in *B. colombiensis* venom [11]. However, some proteins in small quantity may be difficult to identify using a proteomic approach.

Transcriptome analysis based on the analysis of expressed sequence tag (ESTs) provides insight into the regulation of snake venom production and catalogues of transcripts including putative new toxins, toxin isoforms, or low abundant toxins that may be difficult to identify by the proteomic approach [12–19]. Also, with advances in bioinformatics and recombinant DNA technology, venom gland transcriptomic data is an excellent tool for understanding the molecular evolution, developing potential resources for antivenom design and novel therapeutic agents, and studying structure–function relationships.

To provide additional insight into the molecular diversity of venom composition, and identify novel and low abundant toxins, we constructed a cDNA library from the venom glands of a single *B. colombiensis* snake. This database provides a primary assembly of transcripts defined from this species and individual specimen, in which several new venom molecules have been recognized, and could be used as a foundation for venomic studies and evolutionary investigation.

## Results and discussion

### Sequencing and assembly results

**Fig. 1:**
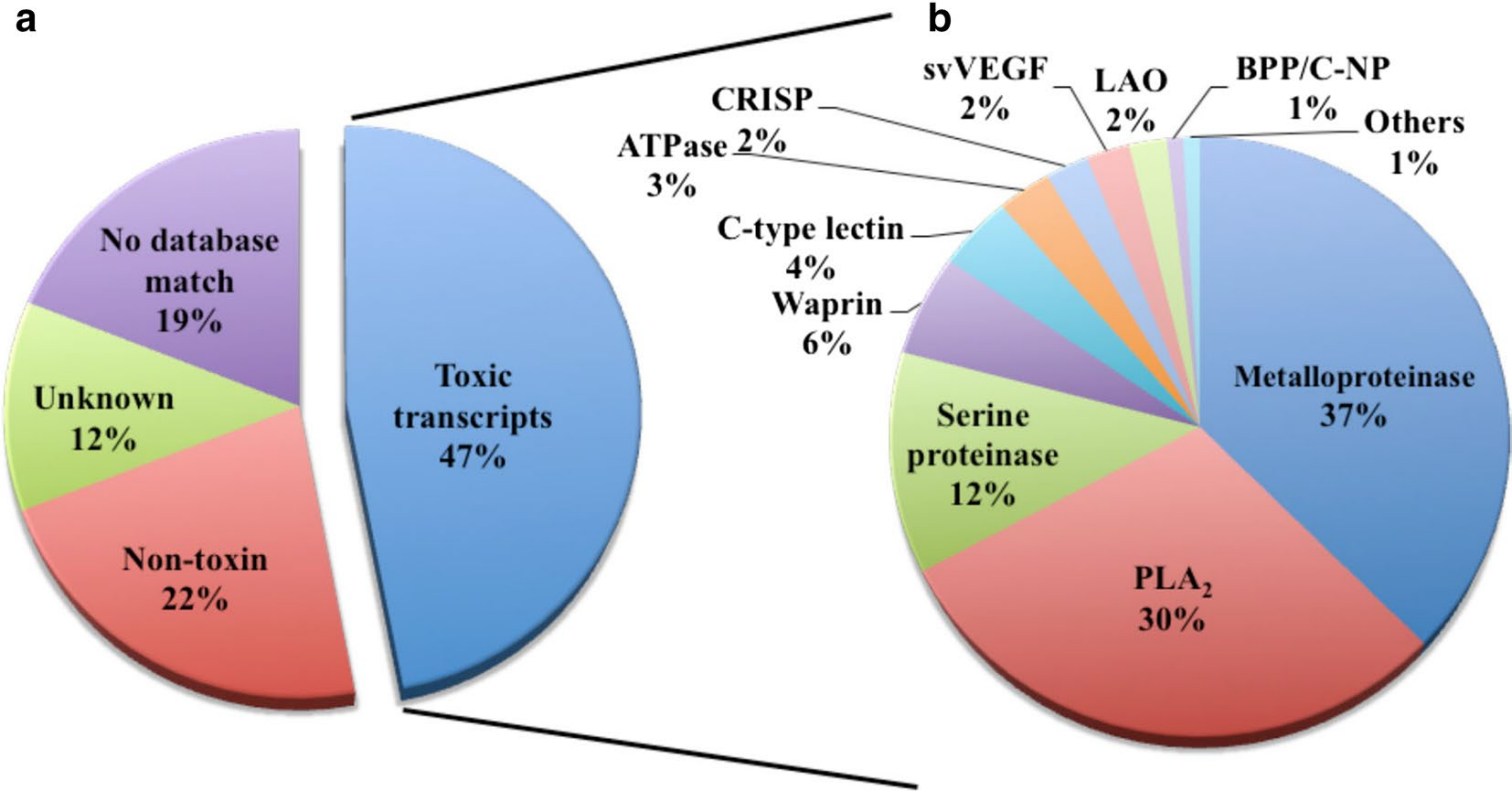
The EST toxin transcripts found in the venom gland of a *B. colombiensis*. The EST toxin transcripts found in the venom gland of a *B. colombiensis*. **a** The *pie graph* shows the relative abundance of all transcripts. Sequences that did not hit anything in the database are indicated as no database match. Unknowns are proteins with no functional attributes. **b** The percentage of the number of transcripts annotated by function terms based on significant BLASTX matches against NCBI GenBank. BLAST only against non-redundant protein sequences (nr) hits with protein. Others represent the minor components with less than three members including phospholipase B (2 ESTs) and phosphodiesterase (1 EST). Details of the individual proteins are shown in Tables 1, 2

The production of this primary cDNA library is an important phase in the upcoming varying field of *Bothrops* gland genomic investigation, gene expression, molecular markers, gene sequencing for structural analyses and possibly for gene screening. Here we constructed a cDNA library from the venom glands of a single snake to preclude ambiguity by intraspecies variation in venom components, which will provide interest in the comparison of the genes expressed among closely related species and within the same species for future work. Therefore, these ESTs may not be representative of all *B. colombiensis*. The cDNA library was generated with a titer of 1.75 × 10^8^ colony-forming units (cfu)/mL. The 2016 clones from the cDNA library were randomly selected and sequenced from the 5′ end. A total of 729 sequences with an average insert size of 593 bp (ranging from 129 to 1265 bp) were obtained after removing low-quality sequences of less than 100 bp and contaminating vector sequences. Sequence analysis revealed that 729 high-quality sequences of which 230 (31.6 %) sequences were singletons and the other 499 (68.4 %) were clustered into 57 contigs assembly by two or more ESTs (Additional file 1). Most of these transcripts matched with snake toxins (344 ESTs in 31 clusters, 47.2 %) as shown in Fig. 1 and Table 1. The other 22 % (160 ESTs in 119 clusters) are classified as non-toxins, which are likely related to cellular process proteins such as ribosomal proteins, translation proteins, regulatory proteins, and elongation factors, and 11.9 % of ESTs (87 ESTs in 64 clusters) are hypothetical proteins with no functional attributes (unknown). The remaining 18.9 % (138 ESTs in 73 clusters) had no hits with any sequences available in the GenBank database. A total number of 108 representative ESTs from an individual cDNA clone were deposited in the NCBI EST database (http://www.ncbi.nlm.gov/dbEST) under accession numbers [dbEST: JZ880059–JZ880166] (Additional file 2).

**Table 1:** Relative abundances of putative toxins identified in *B. colombiensis* venom gland transcriptome

Toxins	No. of ESTs	No. of clusters	Redundancy (clones/clusters)	% Of total	% Of total toxin transcripts
Metalloproteinase	129	10	12.9	17.7	37.5
PLA_2_	102	3	34.0	14.0	29.7
Serine proteinase	41	2	20.5	5.6	11.9
Waprin	19	1	19.0	2.6	5.5
C-type lectin	14	4	3.5	1.9	4.1
ATPase	10	2	5.0	1.4	2.9
CRISP	8	2	4.0	1.1	2.3
svVEGF	8	1	8.0	1.1	2.3
LAO	7	2	3.5	1.0	2.0
BPP/C-NP	3	1	3.0	0.4	0.9
Phospholipase B	2	2	1.0	0.3	0.6
Phosphodiesterase	1	1	1.0	0.1	0.3
Total	344	31	–	47.2	100.0

*BPP/C*-*NP* Bradykinin-potentiating and C-type natriuretic peptide, *CRISP* cysteine-rich secretory protein, *LAO* L-amino acid oxidase, *PLA*_*2*_ phospholipase A_2_, *svVEGF* snake venom vascular endothelium growth factor

**Fig. 2:**
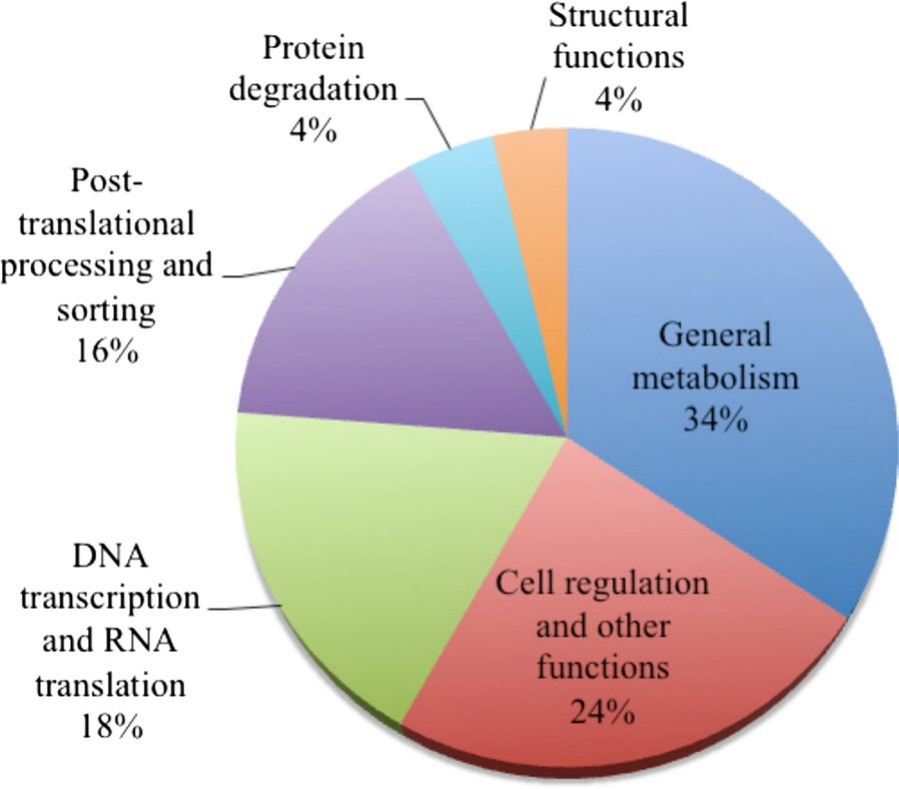
The putative cellular protein transcripts (non-toxins) from *B. colombiensis* according to their cellular functions

The 344 putative toxin-coding ESTs were further clustered and classified into 12 different toxin families using BLAST for functional annotation. These EST clusters coding for the main toxins are listed in Table 2. The most frequent transcripts coding for toxins were from metalloproteinases, followed by phospholipase A_2_s (PLA_2_s) and serine proteinases, which accounted for 79.1 % of the total toxin ESTs. The preponderance of these proteins was expected, as this snake belongs to the genus *Bothrops*, which contain venom most notable for local tissue damage such as edema, hemorrhage, and necrosis [8, 20–23]. Notably, experimental examples of these activities of the *B. colombiensis* venom supporting our findings have been published in the literature [1–3, 8]. However, the toxin transcript expression levels among *Bothrops* species were varied in their relative proportions (Fig. 2 and Additional file 3), which may explain relevant differences observed in the venom action of the species of *Bothrops*.

**Table 2:** Relative abundances of putative toxin-encoding clusters identified in *B. colombiensis* venom gland transcriptome

Clusters	No. ESTs	HIT ID	Annotation	E value
Metalloproteinases				
BC01	107	P83512.2	Snake venom metalloproteinase BaP1 [*Bothrops asper*]	0.00E+00
BC02	1	ABP48735.1	Nonhemorrhagic metalloprotease MP-II, partial [*Bothrops jararacussu*]	9.80E−27
BC03	2	ADO21506.1	MP_IIb1 SVMP precursor, partial [*Bothrops neuwiedi*]	0.00E+00
BC04	1	Q7SZD9.1	Zinc metalloproteinase/disintegrin ussurin [*Gloydius ussuriensis*]	4.40E−21
BC05	5	Q98UF9.3	Zinc metalloproteinase-disintegrin-like HF3 [*Bothrops jararaca*]	0.00E+00
BC06	7	Q2LD49.1	Zinc metalloproteinase-disintegrin-like TSV-DM [*Trimeresurus stejnegeri*]	1.30E−75
BC07	3	C5H5D4.1	Zinc metalloproteinase-disintegrin-like batroxstatin-3 [*Bothrops atrox*]	4.00E−56
BC08	1	Q98UF9.3	Zinc metalloproteinase-disintegrin-like HF3 [*Bothrops jararaca*]	0.00E+00
BC09	1	Q8UVG0.1	Zinc metalloproteinase-disintegrin-like berythractivase [*Bothrops erythromelas*]	0.00E+00
BC10	1	C9E1S0.1	Zinc metalloproteinase-disintegrin-like VMP-III [*Agkistrodon piscivorus leucostoma*]	1.00E−14
Total ESTs	129			
PLA_2_s				
BC11	71	P24605.3	Basic phospholipase A_2_ homolog 2, myotoxin II [*Bothrops asper*]	3.00E−88
BC12	1	P20474.2	Basic phospholipase A_2_ myotoxin III [*Bothrops asper*]	5.30E−13
BC13	30	5.40E−82	Acidic phospholipase A_2_ BmooPLA_2_ [*Bothrops moojeni*]	5.40E−82
Total ESTs	102			
Serine proteinases				
BC14	39	P04971.1	Thrombin-like enzyme batroxobin [*Bothrops atrox*]	3.50E−146
BC15	2	Q5W959.1	Snake venom serine protease HS114 [*Bothrops jararaca*]	5.90E−109
Total ESTs	41			
Waprin				
BC16	19	BAN89446.1	Waprin, partial [*Ovophis okinavensis*]	1.0E−17
Total ESTs	19			
C-type lectins				
BC18	8	P0C930.1	Snaclec bothroinsularin subunit beta [*Bothrops insularis*]	2.20E−49
BC19	3	BAN89423.1	C-type_lectin_beta-subunit [*Ovophis okinavensis*]	1.00E−12
BC20	2	BAP39929.1	C-type lectin B subunit, partial [*Protobothrops elegans*]	3.60E−38
BC21	1	BAP39964.1	C-type lectin F IX/X B [*Protobothrops flavoviridis*]	5.5E−79
Total ESTs	14			
ATPase				
BC22	9	ACJ46370.1	ATPase 6 [*Agkistrodon piscivorus*]	2.70E−93
BC23	1	ETE73855.1	V-type proton ATPase subunit e 1 [*Ophiophagus hannah*]	9.70E−47
Total ESTs	10			
CRISP				
BC24	7	BAP39957.1	Cysteine-rich venom protein, partial [*Protobothrops flavoviridis*]	1.10E−158
BC25	1	ETE59024.1	Cysteine-rich protein 1 [*Ophiophagus hannah*]	6.60E−49
Total ESTs	8			
svVEGF				
BC26	8	Q90X24.1	Snake venom vascular endothelial growth factor toxin [*Bothrops insularis*]	3.60E−93
Total ESTs	8			
LAO				
BC27	6	B5AR80.1	L-amino-acid oxidase [*Bothrops pauloensis*]	0.00E+00
BC28	1	X2JCV5.1	L-amino-acid oxidase [*Cerastes cerastes*]	5.30E−39
Total ESTs	7			
BPP/C-NP				
BC29	3	BAP39952.1	Bradykinin-potentiating and C-type natriuretic peptides, partial [*Protobothrops flavoviridis*]	2.00E−09
Total ESTs	3			
Phospholipase B				
BC30	1	BAN82026.1	Phospholipase B [*Protobothrops flavoviridis*]	3.70E−152
BC31	1	F8S101.1	Phospholipase B [*Crotalus adamanteus*]	3.60E−107
Total ESTs	2			
Phosphodiesterase				
BC32	1	BAN89425.1	Phosphodiesterase [*Ovophis okinavensis*]	0.00E+00
Total EST	1

### Major toxins

#### Metalloproteinases

The highest number of toxin ESTs in *B. colombiensis* were metalloproteinases (37.5 %). This abundance of metalloproteinases has been already observed for other *Bothrops* transcriptomes. The percentages for these reported metalloporteinases range, approximately, between 25–80 % and the highest reported was for *B. alternatus* (urutu) representing 81.4 % of the toxins transcripts [24]. Other *Bothrops* with a high expression of metalloproteinase genes were *B. atrox* (61.6 %) [25] and *B. jararaca* (29.9–53.1 %) [26, 27] (Fig. 2; Additional file 3).

Metalloproteinases are crucial components in hemostasis as well as in thrombosis [28]. Snake venom metalloproteinases (SVMPs) are responsible for the hemorrhagic condition, which is one of the most severe consequences of Viperidae snake envenomations. SVMPs are classified into three subclasses established on their domain structure [29, 30]. These SVMP groups are: The P-I class (20–30 kDa) comprises a single metalloproteinase domain. The P-II class (30–60 kDa) involves a metalloproteinase domain and a disintegrin domain. The P-III class (60-100 kDa) comprises a metalloproteinase, disintegrin-like and cysteine-rich domains [31]. The former P-IV class, a P-III structure which includes an additional C-type lectin-like domain was re-classified into a P-IIId subclass.

When a blood vessel is damaged by SVMPs, these circulating enzymes adhere and accumulate on the disrupted surface of the subendothelium and activate platelets. The aggregation and adhesion of these cells to the subendothelium are facilitated through the interaction of extracellular matrix proteins with their agonist receptors, namely integrins, on the platelet membrane [32, 33]. This intraluminal cell adhesion may initiate the athero-thrombotic process leading to intravascular thrombosis [34–36]. On the other hand, snake venom hemorrhagic metalloproteinases can also digest several blood coagulation components, counting fibrinogen and von Willebrand factor, which amplify the hemorrhagic activity [20, 37]. As described above, the disintegrin domain is part of snake venom metalloproteinases, and mostly derived by proteolytic processing of the protein precursor to produce a free disintegrin [38–40]. Disintegrins are low molecular weight proteins ranging from 49–84 amino acids in length that are known to be involved in cell adhesion ligand recognition, binding specifically to integrin receptors on the cell surface and also exhibiting anti-platelet aggregation activity. Because of their low molecular weight and ability to block integrin activity, both native and recombinant disintegrins have been widely investigated for their anti-cancer activities in biological systems in vitro and in vivo [41–48].

**Fig. 3:**
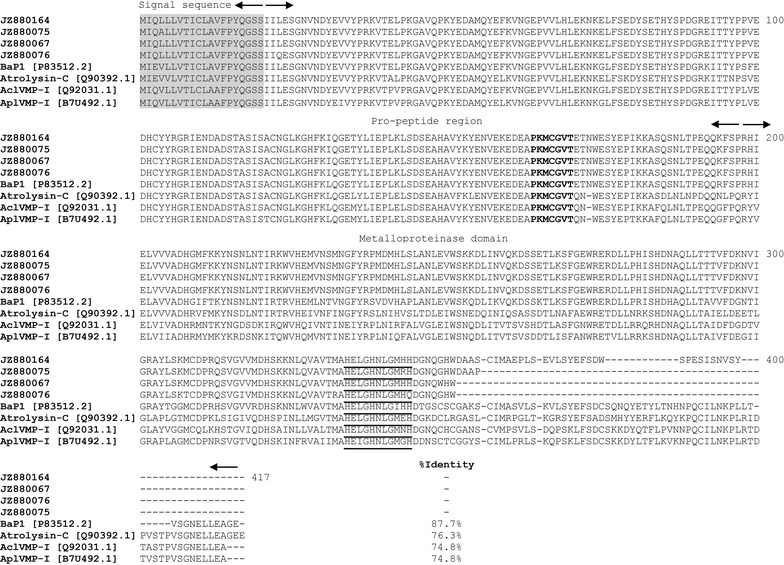
The partially deduced amino acid sequence of the representative clones [JZ880164, JZ880075, JZ880067, and JZ880076] from the most abundant PI-SVMP cluster BC01. Predicted amino acid sequences of transcripts coding for the signal peptide, pro-peptide region, and partial a metalloproteinase catalytic domain with a zinc-binding motif. The signal peptide is highlighted in *grey color*, and the cysteine-switch motif (PKMCGVT) is *bolded*. The zinc-binding site is *underlined*. Each domain is indicated by *arrows*. The major isoform of cluster BC01 is aligned with BaP1 [P83512.2] from *B*. *asper*, atrolysin-C [Q90392.1] from *C. atrox*, AclVMP-I [Q92031.1] from *A. contortrix laticinctus*, and AplVMP-I [B7U492.1] from *A. piscivorus leucostoma*. The  % identity is shown in the figure

In the current work, we identified 129 putative metalloproteinases, which were grouped into five contigs and five singletons (Fig. 1, Tables 1, 2). These 10 unique SVMP clusters corresponding to partial cDNA sequences were named BC01–BC10. The *B. colombiensis* transcriptome contained transcripts for P-I (83.3 % of the total SVMP ESTs and 31.4 % of total toxin transcripts, clusters BC01 and BC02), P-II (2.3 % of the total SVMP ESTs and 0.9 % of total toxin transcripts, BC03 and BC04), and P-III (14 % of the total SVMP ESTs and 5.2 % of total toxin transcripts, BC05–BC10). The BC01 cluster showed the most abundant metalloproteinase cluster (83 % of the total SVMP ESTs and 31.1 % of total toxin transcripts). It posesses a 20-residue signal sequence, a conserved M12B propeptide region, and a reprolysin domain with a zinc-binding motif (HEXXHGXXH motif). NCBI BLAST analysis showed that the deduced amino acid sequence of the partial PI-SVMP cluster BC01 had 87.7 % sequences identity with metalloproteinase BaP1 (P-I class) [P83512.2] from *B. asper*, atrolysin-c [Q90392.1] from *C. atrox* with 76.3 % identity, and was 74.8 % identical to AclVMP-I [Q92031.1] from *Agkistrodon contortrix laticinctus* and AplVMPI [B7U492.1] from *A. piscivorus leucostoma* (Fig. 3). In addition, BC02 (0.3 % of total toxins) were homologous to a non-hemorrhagic MP-II (BjussuMP-II) from *B. jararacussu* venom gland. According to Marcussi et al. [49], BjussuMP-II isolated from *B. jararacussu* snake venom belongs to class P-I devoid of hemorrhagic activity, but exhibit proteolytic activity on some components of the extracellular matrix proteins such as fibrinogen, collagen, and gelatin. Additionally, several non-hemorrhagic P-I SVMPs with fibrino(geno)lytic activity have been reported in *Bothrops* species such as colombienase-1 and colombienase-2 from *B. colombiensis* [2], BJ-PI2 from *B. jararaca* [50], and neuwiedase from *B. neuwiedi* [51].

**Fig. 4:**
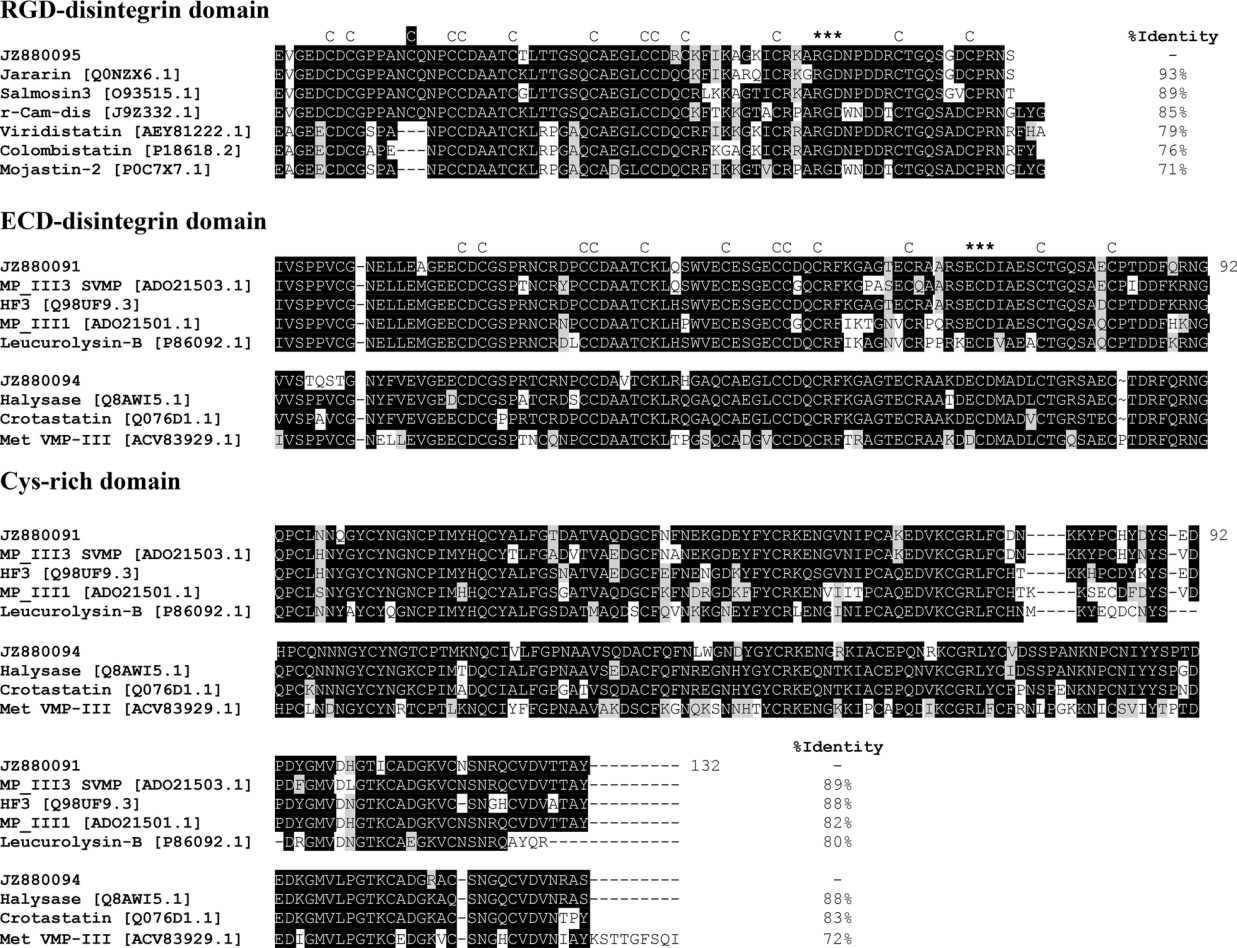
The multiple sequence alignments of the RGD-disintegrin domain (cluster BC03, a representative clone JZ880095), ECD-disintegrin and Cys-rich domains (cluster BC05, a representative clone JZ880091, and cluster BC06, a representative clone JZ880094) of metalloproteinases predicted from partially sequenced clones from *B. colombiensis* with other homologous venom proteins. The major isoforms from each group is aligned with closely related protein in the database, and the  % identities are shown in the figure. The alignment was generated with the ClustalW multiple sequence alignment program with manual adjustment and displayed with shaded boxes. The numbers in parenthesis are the NCBI accession numbers. The *asterisk* above the sequences represent the tripeptide binding motif in PII (RGD) and in PIII (ECD). All cysteine residues (letter “C” above the sequences) are conserved except for an extra cysteine residue (the letter “C” highlighted in *black*) in JZ880095, JZ8800100, jararin, salmosin 3, and r-Cam-dis. The source of sequences were as follows: Jararin [Q0NZX6.1] from *B. jararaca*, r-Cam-dis [J9Z332.1] from *C. adamanteus*, salmosin-3 [O93515.1] from *G. brevicaudus*, viridistatin [AEY81222.1] from *C. viridis viridis*, mojastin 2 isolated from the venom of *C. scutulatus scutulatus*, the native disintegrin colombistatin [P18618.2] from *B. colombiensis*, MP_III3 SVMP [ADO21503.1] from *B. neuwiedi*, HF3 from *B. jararaca*, MP_III1 [ADO21501.1] from *B. neuwiedi*, leucurolysin-B [P86092.1] from *B. leucurus*, Halysase [Q8AWI5.1] from *Gloydius halys*, Crotastatin [Q076D1.1] from *C. durissus terrificus*, and Met VMP-III [ACV83929.1] from *A. contortrix laticinctus*

The BC03 belongs to the P-II metalloproteinases and are all 5′-truncated transcripts with a region of Zn^2+^ binding site and RGD-disintegrin domain, a main integrin receptor-binding motif. We compared the RGD-disintegrin region of the representative clone [JZ880095] with other closely related medium-sized disintegrins, which was a homolog to Jararin [Q0NZX6.1] from *B. jararaca*, r-Cam-dis [J9Z332.1] from *C. adamanteus*, salmosin-3 [O93515.1] from *Gloydius brevicaudus*, viridistatin [AEY81222.1] from *C. viridis viridis*, mojastin 2 isolated from the venom of *C. scutulatus scutulatus*, the native disintegrin colombistatin [P18618.2] from *B. colombiensis* with 93, 89, 85, 79, 76, and 71 %, respectively (Fig. 4). A singleton BC04 is a partial sequence with an RGD motif and had 95.7 % identity with the RGD-disintegrin domain of a metalloproteinase identified in the cDNA library from the venom gland of *G. ussuriensis* [52].

The clusters BC05-BC10 are all class P-III metalloproteinases containing Zn^2+^ binding motifs, the diversity among PIII isoforms disintegrin-like (D/SECD motif), and cysteine-rich domains. We also compared the deduced amino acid sequences from the ECD-disintegrin and Cys-rich domains (Fig. 4). High partial sequence identity (72–89 %) was observed between the major isoforms from the most abundant PIII-SVMP clusters BC05 [JZ880091] and BC06 [JZ880094] and were compared with other viperid SVMPs (Fig. 4).

In the venom-gland transcriptome of *B. colombiensis,* there is a large quantity of class P-I SVMPs (with some of P-III and few P-II SVMPs), contributing to its moderate venom toxicity [1–3], which is relative to the snake’s large size and high production of venom. Our expression profile of SVMPs is in accordance with the proteomic studies as previously reported by Calvete et al. [11] (Table 3), which demonstrated that the P-I SVMPs (30.8 % of total toxins) were the major proteins expressed in the venom of *B. colombiensis* with few P-III (11.3 %). No P-II SVMPs were detected by proteomic analysis, however, a medium-sized disintegrin (5.6 %) was found and was similar to colombistatin (RGD-disintegrin) as previously reported by Sánchez et al. [46]. The absence of P-II SVMPs with few disintegrins and disintegrin-like/cysteine-rich (DC) domain of P-III SVMPs in snake venom protein but not in our transcriptome, it could be due to the post-translational processing of disintegrin domain and DC fragment from its precursor form of the P-II and P-III classes, respectively [38, 53, 54].

**Table 3:** The proportional representation of each *B. colombiensis* venom protein family as predicted from the venom grand transcriptome (this study) and venom proteomics as previously reported by Calvete [11]

Protein family	% Total venom gland predicted toxin transcripts	% Total venom proteins
Metalloproteinase	37.5	42.1
PI	31.4	30.8
PII	0.9	–
PIII	5.2	11.3
PLA_2_	29.7	44.3
PLA_2_-K49	20.9	34.1
PLA_2_-D49	8.7	10.2
Serine proteinase	11.9	<1
WAP	5.5	–
C-type lectin	4.1	–
Nucleotidases	3.2	–
CRISP	2.3	0.1
svVEGF	2.3	–
LAO	2	5.7
BPP/C-NP	0.9	0.8
Phospholipase B	0.6	–
Medium disintegrin	–	5.6
DC fragment^a^	–	0.5

^a^Disintegrin-like/cysteine-rich fragment

In our group, there have been substantial commitments to propose venom metalloproteinases and disintegrins as therapeutic agents acting on hemostasis [1–3, 8, 46, 55–61]. Because of proteomic limitations, the development of cDNA libraries could expedite the use of snake venom metalloproteinases and disintegrins as therapeutics against thrombus formation.

#### Phospholipases A_2_ (PLA_2_)

Phospholipase A_2_ (PLA_2_) is one of the major components in Viperidae snake venoms responsible for the induction of local tissue damage. PLA_2_ enzymes are known to exhibit distinct pharmacological effects, such as local myonecrosis, lymphatic vessel damage, cytotoxicity, anticoagulant, hemolysis, and hemorrhage [62, 63].

PLA_2_ (29.7 % of total toxins) was the second most highly expressed toxin component of the *B. colombiensis* transcriptome. In general, after *B. jararacussu* [64], the observed PLA_2_ transcript abundance from *B. colombiensis* is one of the highest described thus far, greater than that reported for *B. asper* (14.2–17.8 %), *B. atrox* (13.3 %), *B. jararaca* (0.7–9.5 %), *B. insularis* (6.7 %), and *B. alternatus* (urutu) (5.6 %) (Fig. 2; Additional file 3) [24–26, 64, 65].

**Fig. 5:**
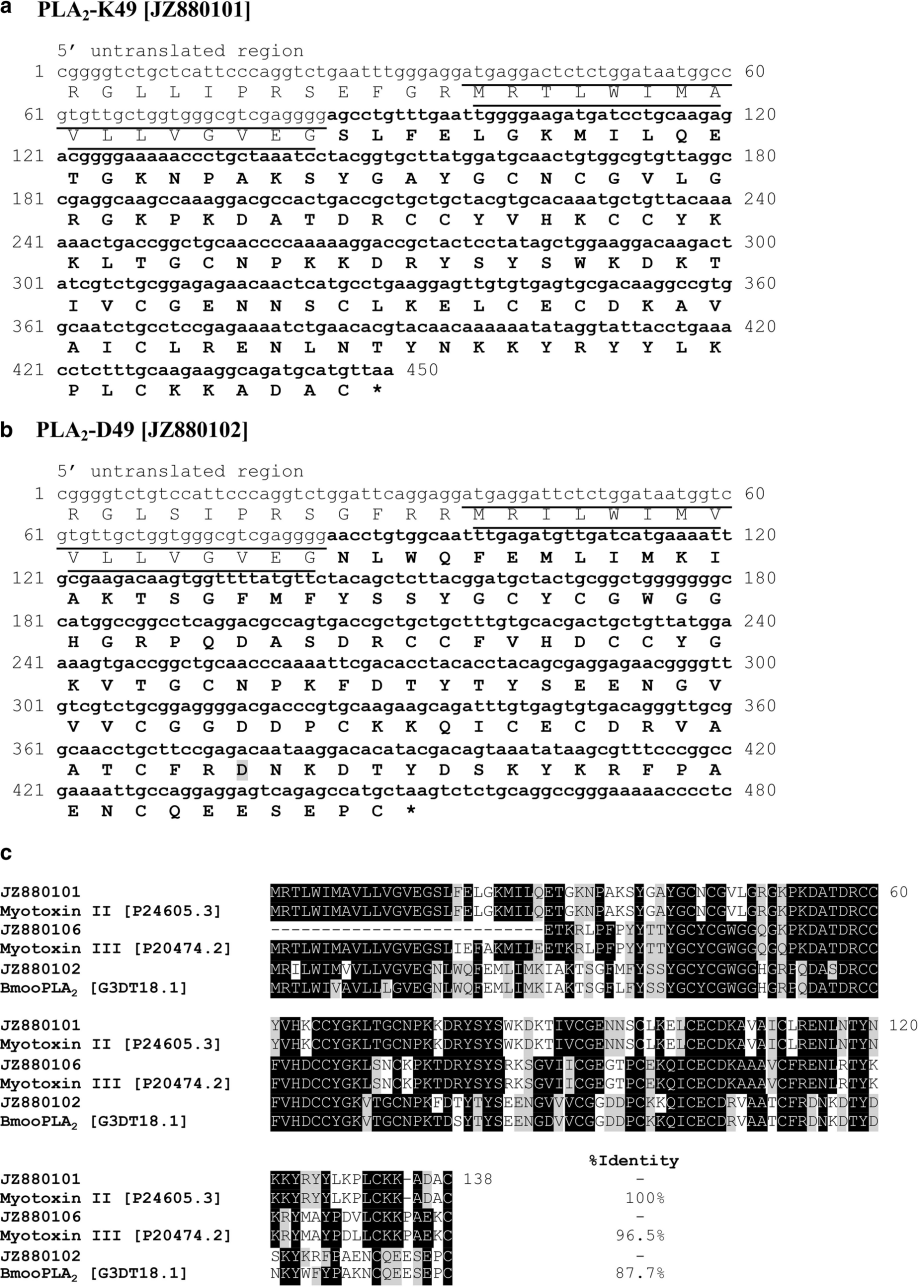
cDNA and deduced amino acid sequences of a representative clone PLA_2_-K49 from cluster BC11 [dbEST: JZ880101] (**a**) and a representative PLA_2_-D49 clone from cluster BC13 [dbEST: JZ880102] (**b**). The 16-residue signal peptide is *underlined*. The mature sequence is *bolded*. (**c**) Multiple alignments of predicted amino acid sequences of PLA_2_-K49, PLA_2_-D49, and basic D49-PLA_2_ with other homologous venom proteins. The alignment was generated with the ClustalW multiple sequence alignment program with manual adjustment and displayed with *shaded boxes*. PLA_2_-K49 [dbEST: JZ880101] is identical to basic the PLA_2_ homolog 2 (myotoxin II) from *B. asper*. Basic D49-PLA_2_ [dbEST: JZ880106] had 96.5 % identity to basic PLA_2_ myotoxin III [P20474.2] from *B. asper*. PLA_2_-D49 [dbEST: JZ880102] is closely homologous to BmooPLA_2_ from *B. moojeni*. The numbers in parenthesis are the NCBI accession numbers

In our database, we obtained two clusters (BC11 and BC13) with full coding sequences and one singleton (BC12). The BC11 showed 100 % similarity to basic PLA_2_ myotoxin II [P24605.3] of *B. asper* with a lysine (Lys) at position 49 (PLA_2_-K49) at the catalytic site, which is commonly found in *Bothrops* species (Fig. 5a, c). The BC13 showed an aspartic acid (Asp) in the same position (PLA_2_-D49) and was matched with an acidic PLA_2_, BmooPLA_2_ [G3DT18.1] of *B. moojeni* with 87.7 % of similarity (Fig. 5b, c). The PLA_2_-K49 was more than twofold abundant than the PLA_2_-D49 (Table 3). This observation corresponded to the proteomic profile reported by Calvete et al. [11].

Interestingly, we found one transcript (BC12) with a 5′ truncated sequence sharing 96.5 % similarity with a basic PLA_2_ myotoxin III [P20474.2] from *B. asper*, and its deduced amino acid sequence showed the Asp49 residue (Fig. 5c). Although two basic Asp49 PLA_2_s have been isolated from *B. jararacussu* (BthTX-II) and *B. pirajai* (PrTX-III), both exhibiting a myotoxic effect [66–68], this is the first basic Asp49 PLA_2_ found in *B. colombiensis.* However, the proteomic work done by Calvete et al. [11] on this same species did not identify this toxin.

#### Serine proteinases

**Fig. 6:**
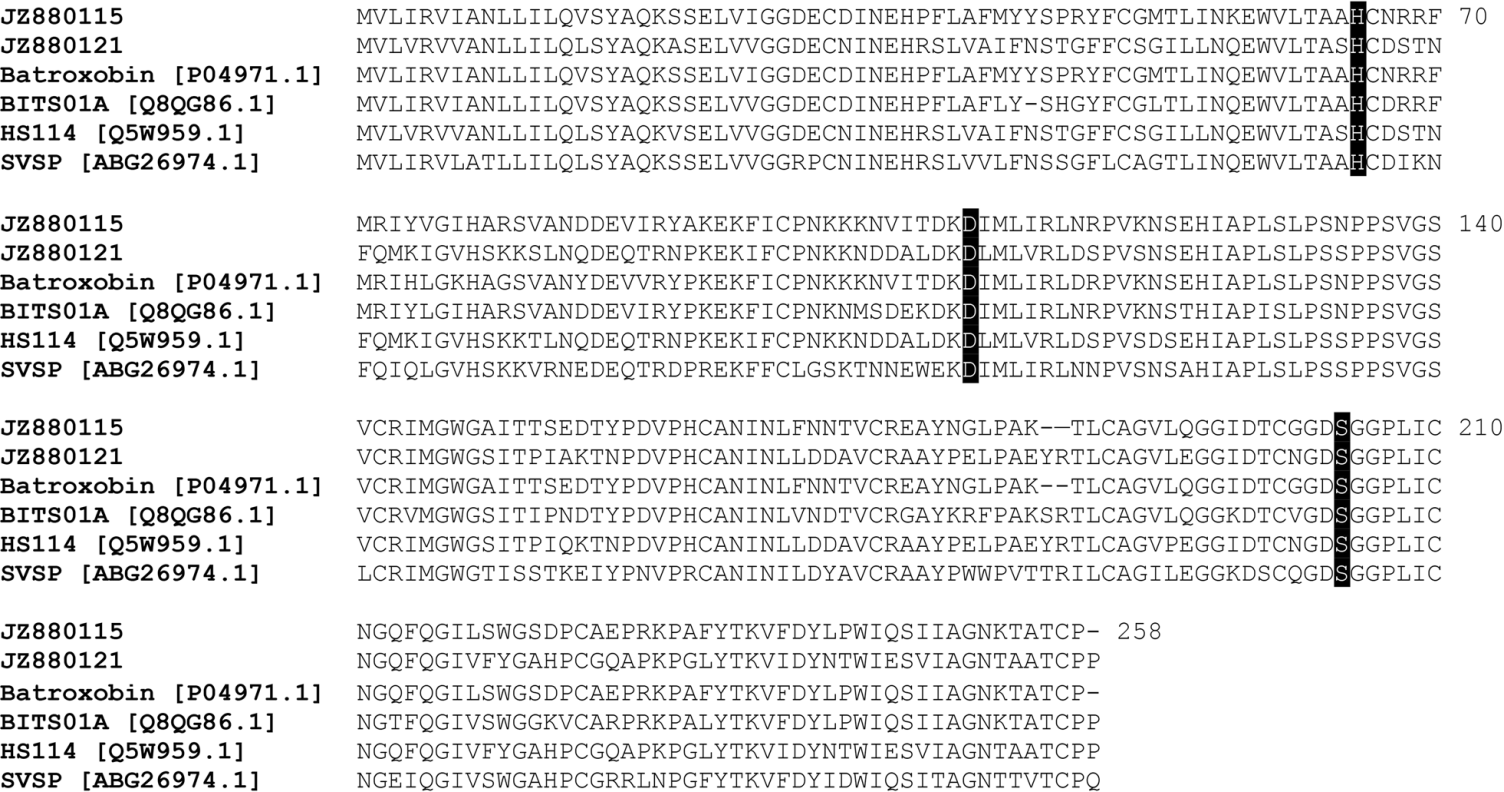
The multiple alignments of completed predicted amino acid sequences of the major isoforms of each cluster BC14 [JZ880115] and BC15 [JZ880121] with batroxobin [P04971.1] from *B. atrox*, BITS01A [Q8QG86.1] from *B. insularis*, HS114 [Q5W959.1], HS114 [Q5W959.1] from *B. jararaca*, and SVSP [ABG26974.1] from *Sistrurus catenatus edwardsii*. Residues forming the catalytic triad are highlighted with a *dark background*

Serine proteinases are abundant and widely distributed in snake venoms and interfere mainly with the hemostatic system [69–71]. We identified two unique, full-length serine proteinases in the cDNA library of *B. colombiensis* and were denoted BC14 and BC15. The BC14 was a major serine proteinase cluster (39 ESTs, 11.3 % of total toxins) sharing 83.9 % identity with thrombin-like enzyme batroxobin from *B. atrox*. The BC15 (2 ESTs, 0.6 % of total toxins) shared a slight similarity (69.6 %) to snake venom serine protease, HS114, from *B. jararaca*. The complete coding sequences of representative clones JZ880115 and JZ880121 from clusters BC14 and BC15; respectively, ranged from 255 to 258 amino acids in length, containing a trypsin-like serine proteinase domain with highly conserved functional residues of the catalytic triad (His41, Asp86, and Ser178 for BC14 and Ser180 for BC15) (Fig. 6). The putative identity matches for each representative clone is shown in Additional file 2.

Overall, they accounted for 11.9 % of the toxin transcripts, which is at least twofold more expressed than that of any other components of the remainder toxin genes in this snake. The abundance of these genes is high among the genus *Bothrops* transcriptomes presented in the literature, and comparable, for example, to the 8.3 % reported in Pacific *B. asper* [65] and 8.1 % in *B. atrox* [25], respectively (Fig. 2; Additional file 3). Our results are also similar to the recently described transcripts for *B. jararaca* (8.1 %) by Zelanis et al. [26]. However, older data published by Cidade et al. [27] for *B. jararaca*, indicates 28.5 % for serine proteinase. Other species of *Bothrops* express considerably less serine proteinase transcripts than *B. colombiensis*, e.g. *B. jararacussu* (2.4 %) and *B. alternatus* (urutu) (1.9 %) [24, 64].

### Other less-abundance toxins

The principal venom components of the *B. colombiensis* transcriptome are metalloproteinases, PLA_2_, and serine proteinases, ranging from 12 to 38 % of the total toxin ESTs. The remainder of the results (the highest expressed is less than 6 %) was categorized in “other less-abundance toxins” because they vary from a relative moderate to low expression level (Table 1).

#### Waprin

From this group of transcripts, Waprin is found to be very interesting. In general, waprin is a small protein containing 50 amino acid residues. This protein belongs to the Whey acidic protein (WAP) family due to its four-disulfide core domain structure. The WAP domain is found in proteins which are highly divergent regarding a broad range of biological functions involving the innate immune system, regulating cell proliferation, and inhibiting various cellular proteins [72, 73]. The function of waprin in snake venom is unknown. Only one waprin from *Oxyuranus microlepidotus* venom has been examined, and it was shown to act as an antimicrobial [72].

**Fig. 7:**
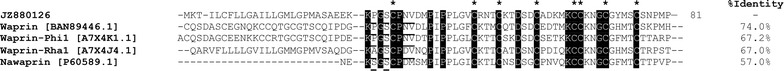
Multiple alignments of the full-length coding sequences of major isoforms of waprin (cluster BC16, a representative clone JZ880126) from *B. colombiensis* with other homologous venom proteins. The alignment was generated with the ClustalW multiple sequence alignment program with manual adjustment and displayed with *shaded boxes*. The numbers in parenthesis are the NCBI accession numbers. Identical residues are marked in *black*. All cysteine residues (an *asterisk* above the sequences) are conserved. The *bar* (-) was introduced for optimal comparison. The inhibition loop is *underlined*. The sources of sequences are as follows: waprin [BAN89446.1] from *Ovophis okinavensis*, waprin-Phi1 [A7X4K1.1] from *Philodryas olfersii*, waprin-Rha1 [A7X4J4.1] from *Rhabdophis tigrinus tigrinus*, Nawaprin [P60589.1] from *Naja nigricollis*. The  % identities are shown in the figure

In this library, 19 waprins were identified and were grouped into 1 cluster named BC16, comprising 5.5 % of total toxins (Tables 1, 2). All waprin sequences are full-length coding sequences with a conserved WAP domain and an inhibitory loop. The protein BLAST search and CLUSTAL W multiple sequence alignments revealed that the amino acid sequence of a representative clone JZ880126 of waprin was homologous to waprin [BAN89446.1] from *Ovophis okinavensis*, waprin-Phi1 [A7X4K1.1] from *Philodryas olfersii*, waprin-Rha1 [A7X4J4.1] from *Rhabdophis tigrinus tigrinus*, and Nawaprin [P60589.1] from *Naja nigricollis* with 74, 67.2, 67, and 57 % identity, respectively (Fig. 7).

Waprin is very uncommon in venom of *Bothrops* snakes. *Bothrops* species express this toxin transcript at a very low level, or even undetectable, but its expression was the fourth most highly expressed transcript in *B. colombiensis* (Additional file 3). In a study of eight snake venom transcriptomes of distinct genera (*Crotalus*, *Bothrops*, *Atropoides*, *Cerrophidion*, and *Bothriechis*) from Costa Rica, waprin ESTs were only and barely detected in *B. asper* (0.1 %) individuals from the Caribbean, but not in *B. asper* from the Pacific region [65]. However, waprin seems to be a transcript frequently recovered from venom transcriptomes in other snakes from other snake families such as Elapidaes (*Naja nigricollis*, *O. microlepidotus*, *Acanthophis wellsi*, and others), Colubridaes (*Thrasops jacksonii*), Dipsadidaes (*Liophis poecilogyrus* and *P. olfersii*), Homalopsidaes (*Enhydris polylepis*), and Natricidae (*Rhabdophis tigrinus*) [74, 75]. This unexpected abundance of waprin genes found in our transcriptome opens new avenues for further investigation.

#### C-type lectins

Snake venom C-type lectins (snacles) are commonly found in snake venoms. They affect blood coagulation and platelet function by interacting with some proteins in the blood coagulation system and cell surface receptors, causing an imbalance of the hemostatic system [76].

We obtained three clusters of partial C-type lectins named BC18–BC20 and one singleton (BC21). The BC18 accounted for 2.3 % of total toxins with 82.7 % similarity to the beta subunit snaclec, bothroinsularin, isolated from *B. insularis* [77]. Clusters BC19 (0.9 % of total toxins) and BC20 (0.6 % of total toxins) were 62 and 69.4 % identical to C-type lectin beta-subunit from *Ovophis okinavensis* and from *Protobothrops elegans*, respectively. The complete sequence (BC21) showed 77.4 % identity to C-type lectin F IX/X B from *Protobothrops flavoviridis*. The deduced amino acid sequence contained 146 amino-acid residues with a carbohydrate-recognition domain and highly conserved seven cysteine residues.

In total, C-type lectins transcripts accounted for 4.1 % of the total toxins, which was higher than that of *B. alternatus* (1.4 %), *B. asper* Pacific (1.3 %) and Caribbean (1 %). However, its abundance was considerably less than in *B. insularis* (14.2–14.5 %), *B. jararaca* (8.3–22.3 %), *B. jararacussu* (7.4 %), and *B. atrox* (6.6 %) (Fig. 2; Additional file 3) [24–27, 64, 65, 78].

#### Nucleotidase

Nucleotidases (5′ nucleotidase, ADPase, ATPase, and phosphodiesterase) are hydrolytic enzymes found in snake venoms that have an important role in the releasing of adenosine from nucleic acids [79, 80]. The generation of adenosine, a multitoxin, could interfere with different biological activities such as inhibiting platelet aggregation, inducing the diffusion of toxins by increasing vascular permeability through vasodilation, and immobilization of prey by depletion of ATP [80–83].

We obtained one cluster (BC22) of ATPase (9 ESTs, 2.6 % of total toxins) and a singleton (BC23, 0.3 % of total toxins). They matched with ATPase six from *A. piscivorus* and V-type proton ATPase subunit e 1 from *Ophiophagus hannah*, and the similarity scores were 79.2 and 96.3 %, respectively. We also found a singleton (BC32, 0.3 % of total toxins) encoding a phosphodiesterase, which showed 81 % identity with a phosphodiesterase from *Ovophis okinavensis*. Several transcriptomic and proteomic studies reported the expression of these toxins in various snakes [24, 65, 84–88]. However, the function of these enzymes during envenomation remains unclear.

#### Cysteine-rich secretory proteins (CRISP)

Eight ESTs sequences of the cysteine-rich secretory proteins (CRISP) were recovered from the *B. colombiensis* cDNA library. CRISP proteins are an evolutionarily conserved family, which possess 16 highly conserved cysteine residues, 10 of these cysteines are located in the carboxyl-terminal third end. These proteins have been found in the mammalian male reproductive tract and in snake venoms and salivary extractions [89–91].

One major CRISP cluster named BC24 (7 ESTs, 2 % of total toxins) and one singleton (BC25, 0.3 % of total toxins) were identified. The complete coding sequence of BC24 showed 83.7 % identity with a partial cysteine-rich venom protein from *P. flavoviridis*. The *B. colombiensis* homolog was 258 amino acids in length and contained two conserved domains including a sperm-coating protein (SCP)-like extracellular protein domain with N-terminal pathogenesis-related protein-1 (PR-1) domain and a C-terminal cysteine rich domain (CRD) with 10 conserved cysteine residues. The singleton BC25 was homologous to a cysteine-rich protein 1 from *O. hannah* with 98.7 % identity. Its deduced amino acid sequence consisted of a 54-residue LIM (lin-11-isl-1-mec-3) domain containing two zinc finger motifs with eight conserved residues, seven cysteines and one histidine.

CRISPs represent the seventh most abundant toxin transcript in *B. colombiensis* venom, 2.3 % of the whole toxin genes. This is one of the highest reported in the literature. The values are under 1 % for *B. alternatus*, *B. atrox*, and *B. asper*. While *B. jararacussu* (1 %), *B. insularis* (0.6–1.5 %), and *B. jararaca* (1–1.6 %) expressed values over this cutoff (Fig. 2; Additional file 3) [24–27, 64, 65, 78]. Interestingly, the only comparable values to our findings are the CRISP transcript level described in the newborn venom glands from *B. jararaca* (2.7 %), which is about two or threefold higher than that of adult [26].

Although the CRISP family is widely distributed in snake venoms, there is scarce information about its contribution to the pathology of snakebites. A CRISP protein from *Philodryas patagoniensis* snake venom was described to cause myonecrosis in a murine model [92]. Recently, Estrella et al. [89] isolated a CRISP protein, helicopsin, from the broad-banded water snake (*Helicops angulatus*), which has been shown to exhibit neurotoxic activity causing rapid death in mice. In general, CRISP proteins are thought to interfere with smooth-muscle contraction by interfering with the Ca^2+^ and K^+^ channels [75, 93]. An effort needs to be done to improve the knowledge of this toxin. On the other hand, these proteins are attractive as therapeutics, as ion channel modulators represent a high potential as pharmacological agents.

#### Snake venom vascular endothelium growth factor (svVEGF)

svVEGFs have been found in *Bothrops* species and act as mediators of vascular permeability, which may be involved in the absorption of venom toxins and hypotension during envenomation [94, 95]. We found only one cluster (BC26 with 8 ESTs, 2.3 % of total toxins) encoding for svVEGF. The BC25 was homologous to a svVEGF from *B. insularis* with 95.2 % similarity. The abundant of svVEGF was less represented than in *B. insularis* (4.3–4.7 %) but higher than in *B. atrox* (0.9 %), *B. alternatus* (0.6 %) and *B. jararaca* (0.2–1.5 %) (Fig. 2; Additional file 3) [24–27, 78].

#### L-amino oxidase (LAO)

LAOs are widely distributed in snake venoms and are responsible for diverse biological activities including hemorrhage, edema, alterations in blood coagulation, activation or inhibition of platelet aggregation, apoptosis, and cytotoxicity [96]. The abundance of LAO transcripts presented in the *Bothrops* venom gland transcriptomes ranges from 0.5–4.2 % (Fig. 2; Additional file 3) [24–27, 64, 65, 78]. In our EST database, LAO accounted for 2.03 % of total toxins, consisting of one cluster (BC27, 6 ESTs) and one partial singleton (BC28). The BC27 and BC28 had 92.8 and 94 % amino acid similarities to the L-amino-acid oxidase of *B. pauloensis* and *Cerastes cerastes*, respectively.

#### Bradykinin-potentiating and C-type natriuretic peptide (BPP/C-NP)

Bradykinin-potentiating peptides (BPPs) are well known to be inhibitors of the angiotensin-converting enzyme and may contribute to venom-induced hypotension [97]. C-type natriuretic peptides (C-NPs) also play a significant role in vascular and cardiac function [98]. Several studies have reported the presence of BPPs/C-NP in snake venom gland, spleen, pancreas, and brain [99, 100].

We identified three partial transcripts encoding BPP/C-NP in the *B. colombiensis* venom gland library (0.9 % of toxin ESTs, BC29) that shared 86 % similarity with a partial BPP/C-NP from *P. flavoviridis*. The percentage of BPP/C-NP transcripts was particularly very low, one of lower reported in the literature (Fig. 2; Additional file 3). The proportion of this gene in venom toxin of *Bothrops* transcriptomes is 6–23 % [19, 24, 26, 27, 65, 78].

#### Phospholipase B

We detected two partial individual singletons encoding phospholipase B (BC30 and BC31), which were 93.1 and 96.9 % identical with phospholipase B from *P. flavoviridis* and *C. adamanteus*, respectively. The phospholipase B accounted for 0.6 % of the toxin-related genes in the *B. colombiensis* transcriptome. Phospholipase B toxins have been documented in the transcriptomes of *C. adamanteus* [86] and *B. atrox* [25]; however, these toxins in *B. atrox* were grouped as a cellular transcript (Fig. 2; Additional file 3). Phospholipases B were also found in the proteomes of *C. adamanteus* [101], *C. viridis viridis* [102], *B. jararaca*, *B. atrox*, *B. jararacussu*, *B. neuweidi*, *B.altenatus*, and *B. cotiara* [103], and Australian elapid snake, *Pseudechis guttatus* [104]. This molecule could be responsible for the hemolytic activity as previously described in several Australian elapid venoms [105–108].

### Identification of cellular transcripts

**Fig. 8:**
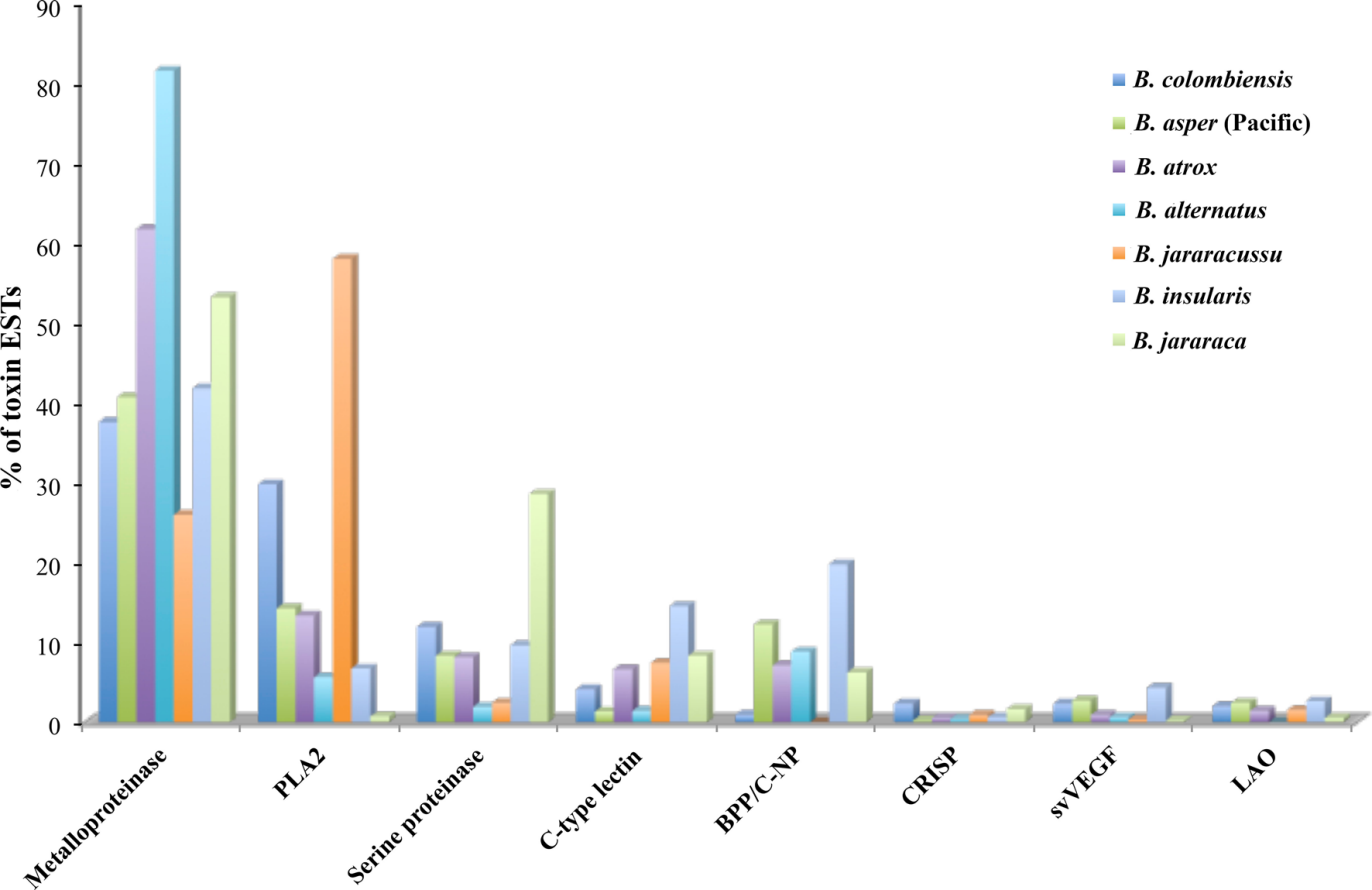
Relative abundance of the major toxin families in *Bothrops* venom gland transcriptomes. The abundance of transcripts is expressed as a percentage of the total toxin transcripts and was calculated by diving the number of ESTs of each toxin family by the total number of toxin ESTs reported in each study. The data sources other than *B. colombiensis* were as follows: *B. asper* (Pacific) [65], *B. atrox* [25], *B. alternatus* [24], *B. jararacussu* [64], *B. insularis* [27], and *B. jararaca* [27]. The percentage of each toxin transcript of individual *Bothrops* species is shown in the Additional file 3

The non-toxin transcripts (160 transcripts) were composed of 22 % of the *B. colombiensis* venom gland. They are categorized into 6 groups according to their biological processes (Fig. 8). The most abundant transcripts coding for metabolic enzymes were related to general metabolism (34.4 % of cellular transcripts and 7.5 % of total transcripts), such as cytochrome c oxidase (21 ESTs, 2.9 % of total transcripts), cytochrome b (11 ESTs, 1.5 % of total transcripts), NADH dehydrogenase (7 ESTs, 1 %). These transcripts are commonly found in snake venom glands [19, 27, 88]. Transcripts related to cell regulation and other functions comprised 23.8 % of cellular transcripts (5.2 % of total transcripts), followed by the DNA transcription and mRNA translation (18.1 % of cellular transcripts and 4 % of total transcripts). Among transcripts for DNA transcription and mRNA translation, we identified mostly ribosomal proteins (11 ESTs, 1.5 % of total transcripts) and elongation factors (5 ESTs, 0.7 % of total transcripts).

The post-translational processing and sorting-related transcripts were accounted for 15.6 % of cellular transcripts and 3.4 % of total transcripts. The most abundant transcripts were parvalbumin (5 ESTs composing only one cluster, 0.7 % of total transcripts), which are calcium-binding proteins belonging to EF-hand protein family. The deduced amino acid sequence of parvalbumin was 97.3 % identical to parvalbumin from *C. oreganus helleri* and had an EF-hand domain with eight highly conserved residues for calcium-binding. We also found one transcript of another calcium binding protein, calnexin. These calcium-binding proteins may be involved in the process of toxin secretion [19, 109]. Other two low abundant house-keeping genes (each 4 % of cellular transcripts) involved in protein degradation including ubiquitin (6 ESTs) and proteasome (1 EST) and six transcripts related to structural functions such as myosin, tubulin, and collagen also were identified.

### Comparison of the transcriptome and proteome of *B. colombiensis*

A proteomic study of *B. colombiensis* was recently published [11], and this allows us to compare the different toxin occurrences between the transcriptome of this current study and proteome of this snake species. It has to be kept in mind that the transcripts are not necessarily synonymous with the protein expression due to regulatory events presents in the translation process from the mRNA to protein. Additionally, the transcriptomic and proteomic approaches, due to their particular methodologies, may favor the presences or absence of a determinate gene or protein. Moreover, the venom used for the proteomic approach was pooled from the Venezuelan regions of Santa Barbara (Barinas State), San Felipe (Yaracuy State), Barlovento and Araira (Miranda State), while the venom glands of a single *B. colombiensis* snake was used for transcriptomic analysis in this study. For these reasons, our integrated comparison of transcriptomic and proteomic data revealed the quantitative differences between the relative occurrences of protein families in the venom gland transcriptome (expressed as a relative number of transcripts) and venom proteome (expressed as the percentage of total HPLC separated proteins) of *B. colombiensis* (Table 3).

The best match was for BPP/C-NP where transcripts and proteins obtained were very similar (Table 3). CRISP presents equally low expression for both protein and gene (0.1 vs. 2.3 %, respectively). Metalloproteinase and PLA_2_ show reasonable agreement between the transcriptome and proteome data. The venom components reported in the proteomic analysis was slightly higher than the transcriptome data for metalloproteinases (42.1 vs. 37.5 %, respectively) and moderate for PLA_2_ (44.3 vs. 29.7 %, respectively). The same was true for the acidic and basic PLA_2_ subclasses: PLA_2_–K49 (34.1 vs. 20.9 %, respectively) and PLA_2_–D49 (10.2 vs. 8.7 %, respectively), even the proportion of subclasses (PLA_2_–K49/PLA_2_–D49) was comparable (3.3-fold for protein vs. 2.4-fold for EST). This was not the case for the third most abundant transcript, serine proteinases comprising of 11.9 %, in which the protein component was less than 1 %. Such difference between transcriptome and proteome occurrences of serine proteinases has already been observed in *B. alternatus* [24].

For the first time, we identified waprin in *Bothrops* spp. transcriptomes, being composed of 6 % of the *B. colombiensis* venom gland but was not detected using a proteomic approach. In addition, common toxins recognized in snake venoms including C-type lectin and CRISP were found in venom transcriptome, accounting for 4.1 and 2.9 %, respectively but was extremely low or undetectable at the protein expression level. These findings showed that the transcriptome and proteome are each other’s confirmatory and complementary approaches to the description of *B. colombiensis* venom.

Symptoms of *B. colombiensis* envenoming include edema, local tissue damage (ecchymoses, blisters, local hemorrhage, and myonecrosis), and thrombocytopenia with increased risk of systemic bleeding from disseminated intravascular coagulation (DIC), cardiovascular shock, and acute renal failure [110]. In agreement with the clinical observations, the venom composition of *B. colombiensis*, based on our transcriptomic and proteomic data [11] (Table 3) is heavily dominated by snake venom components affecting hemostasis including SVMPs, PLA_2_, and serine proteinases. The relative contributions of the major toxin classes in the *Bothrops* snake venom gland transcriptomes revealed the diversity of toxin expression (Fig. 8; Additional file 3). However, the primary toxins including SVMPs, PLA_2_, serine proteinases, BPP/C-NP, and C-type lectins are likely to be categorized in the most abundant toxin groups in most *Bothrops* species. These major toxins are responsible for local and systemic effects by inducing hemorrhage (SVMPs), affecting hemostasis (serine proteinases, C-type lectins, disintegrin, and PLA_2_), myonecrosis (myotoxic PLA_2_), and cardiovascular actions (SVMPs, serine proteinases, PLA_2_, BPP/C-NP) [111–113].

## Conclusion

Snake venoms have a massive impact on human populations through the morbidity and mortality related with snakebites and could also be excellent sources of novel molecules with potential medical applications. In this study, we present the EST database of an individual *B. colombiensis* venom gland, which provides information about the gene expression in a specific specimen and allows a comparative view with the previous proteomic study of this snake. We found many unique toxin sequences, multigene toxin families, and a number of molecules previously not known to be expressed in *B. colombiensis* venom, such as waprin and calcium-binding proteins. However, venom gland transcriptomes based on cloning technologies and random clone selection sequencing may not allow for discovery of rare transcripts due to the recurrent sequencing of more abundant cDNA. Transcriptome analysis using high-throughput RNA-sequence (RNA-seq) would greatly expands the potential for rare transcript discoveries and would provide a much more comprehensive analysis. Our database constitutes the first reference collection of ESTs from *B. colombiensis*. This EST database not only facilitated a better understanding of the pathophysiological effects after envenomation, but also provides a valuable resource for studying structure–function relationships and developing new research tools and therapeutic agents.

## Methods

### Venom gland sample collection

A healthy, 5 years old male *B. colombiensis* originating from Venezuela and housed at the National Natural Toxins Research Center Serpentarium was sacrificed (CO_2_), its venom gland excised and immediately frozen (2 g) in liquid nitrogen and stored until used for RNA isolation. Venom was extracted from the snake 4 days prior to sacrificing. The protocol was approved by the IACUC Texas A and M University-Kingsville, Texas, USA.

### Total RNA isolation and cDNA library construction

Venom glands (10 mg) were disrupted with a pestle and mortar in liquid nitrogen, and total RNA was isolated using the NucleoSpin^®^ RNAII kit (Clontech Laboratories, Inc., CA, USA) based on the company’s instruction. DNA contamination was removed by an on-column rDNase digestion during the preparation. We recovered 1.4 µg of total RNA from 10 mg tissue. The 260/280 absorbance ratios of the total RNA sample was 2.22, indicating purity of the total RNA. The integrity of total RNA was checked by discerning the 28S and 18S bands of ribosomal RNA in 1.2 % agarose gel by staining with ethidium bromide. The 28S/18S RNA bands showed an intensity ratio of about 2:1 that was considered good quality RNA (data not shown). To determine the quantitative and qualitative (RNA quality index, RQI) of the total RNA, the sample was also run through an Experion RNA Analysis Kits using Experion™ Automated Electrophoresis System (Bio-Rad Laboratories, Inc., USA) and the RQI was 7.4, confirming a high-quality RNA sample to be used for library construction. The RQI score is based on a numbering system from 1 to 10 (in ascending quality). In general, an RQI higher than seven represents an acceptable quality of RNA. A directional cDNA library using 120 ng of total RNA was constructed using the In-Fusion^®^ SMARTer™ cDNA Library Construction Kit (Clontech Laboratories, Inc.), which was modified from Suntravat et al. [55]. Briefly, a 120 ng of total RNA from the venom gland was reverse transcribed to the first-strand cDNA using the SMARTScript™ Reverse Transcriptase (Clontech Laboratories, Inc.) and the In-Fusion SMARTer CDS primer (Clontech Laboratories, Inc.) at 42 °C for 90 min. Then, double-stranded cDNA (ds cDNA) synthesis was performed on an iCycler Thermal Cycler (Bio-Rad Laboratories, Inc., CA, USA) by LD PCR reaction containing 73 μL of deionized H_2_O, 10 μL of 10X Advantage 2 PCR buffer, 10 μL of first-strand cDNA, and 2 μL of 50X dNTP Mix, 5′ PCR primer II A, 3′ In-Fusion SMARTer PCR Primer, and 1 μL of 50X Advantage 2 Polymerase Mix. The final volume was 100 μL. PCR conditions included an initial denaturation step at 95 °C for 1 min followed by 18 cycles at 95 °C for 15 s, at 65 °C for 30 s, and at 68 °C for 6 min. Lastly, the ds cDNA was purified using CHROMA SPIN™ + TE-1000 size exclusion column chromatography (Clontech Laboratories, Inc.). Three microliters of each fraction were electrophoresed on a 1.1 % agarose/EtBr gel to determine the peak fractions by visualizing the intensity of the bands under UV. Fractions containing large-, medium-, and small-sized cDNA were pooled, which was ligated into the pSMART2IFD vector (Clontech Laboratories, Inc.). The resulting ligation reactions were transformed into Stellar™ Electrocompetent *Escherichia coli* HST08 strain (Clontech Laboratories, Inc.). The final resulting plasmid library had over 1 million independent clones. The cDNA library constructed is a non-normalized primary library without amplification, so the clone abundance represents the relative mRNA population.

### Plasmid preparation and DNA sequencing

Individual white colonies were randomly selected from a Luria–Bertani (LB) agar plate containing 100 µg/mL ampicillin, 1 mM IPTG, and 75 µg/mL X-Gal and inoculated in 5 mL of LB and 100 µg/mL ampicillin medium overnight at 37 °C with shaking at 225 rpm on an Innova^®^ 43 incubator shaker (New Brunswick Scientific, CT, USA). Plasmid DNAs were isolated using the GenElute™ plasmid miniprep kit (Sigma-Aldrich, MO, USA), according to the manufacture’s instruction. Extracted plasmid DNAs were sent out for automated sequencing at the DNA Facility, Office of Biotechnology, Iowa State University, Iowa. All of the cDNA sequences were 5′ sequenced using the forward screening primer (5′-TCACACAGGAAACAGCTATGA-3′).

### Assembly and identification of ESTs

#### Sequencing analysis

Sequence chromatograms were processed to remove low quality sequences and poly A^+^ tracts using the Lasergene 12 software (DNASTAR, Inc., Madison, WI). Adapter and vector sequences were then removed using the NCBI VecScreen (http://www.ncbi.nlm.nih.gov/tools/vecscreen/). The processed EST sequence files were assembled into contiguous clusters (including contigs and singletons) using the Lasergene 12 software (DNASTAR). Each EST was searched against the non-redundant database of NCBI using BLASTN and BLASTX algorithms to identify similar sequences with an e-value cutoff <10^−5^ and a minimum coverage of 100 bp [87]. Representative 108 cDNA sequences were submitted to the dbESTs division of GenBank under accession numbers [dbEST: JZ880059–JZ880166].

## Additional files

10.1186/s12867-016-0059-7 Summary of statistics after clustering and assembly of 729 EST sequences.
10.1186/s12867-016-0059-7 A representative list of putative protein identity matches for expressed sequence tags (ESTs) obtained from randomly sequenced clones from *B. colombiensis* venom gland.
10.1186/s12867-016-0059-7 Relative contributions of the different putative toxins hits in each of the *Bothrops* snake venom gland transcriptome.

## Abbreviations

BPP/C-NP: Bradykinin-potentiating and C-type natriuretic peptide
CRISP: cysteine-rich secretory protein
CRD: cysteine-rich domain
EST: expressed sequence tag
LAO: L-amino acid oxidase
LIM: lin-11-*Caenorhabditis elegans* cell lineage gene, isl-1-rat insulin I gene enhancer region-binding protein, mec-3-*C. elegans* gene required for differentiation of mechanosensory neurons
PLA_2_: phospholipase A_2_
PR-1: pathogenesis-related protein-1
SCP: sperm-coating protein
svVEGF: snake venom vascular endothelium growth factor
WAP: whey acidic protein
