# Erratum to: Gene expression profiling of the venom gland from the Venezuelan mapanare (*Bothrops colombiensis*) using expressed sequence tags (ESTs)

**DOI:** 10.1186/s12867-016-0062-z

**Published:** 2016-05-24

**Authors:** Montamas Suntravat, Néstor L. Uzcategui, Chairat Atphaisit, Thomas J. Helmke, Sara E. Lucena, Elda E. Sánchez, Alexis Rodríguez-Acosta

**Affiliations:** Department of Chemistry, National Natural Toxins Research Center, Texas A and M University-Kingsville, Kingsville, USA; Laboratorio de Inmunoquímica y Ultraestructura, Instituto Anatómico de la Universidad Central de Venezuela, Caracas, Venezuela

## Erratum to: BMC Molecular Biol (2016) 17:7 DOI 10.1186/s12867-016-0059-7

Unfortunately, after publication of this article [1], it was noticed that two errors were introduced during the production process. The title had the words “venomgland” incorrectly affixed together. The correct term should be “venom gland” as two separate words. Further, figure 8 (Fig. 1 here) and figure 2 (Fig. 2 here) were incorrectly exchanged. The figure legends remain in the same place. Both corrected figures with their associated legends can be found below. The original article has also been updated to correct these errors.

**Fig. 1:**
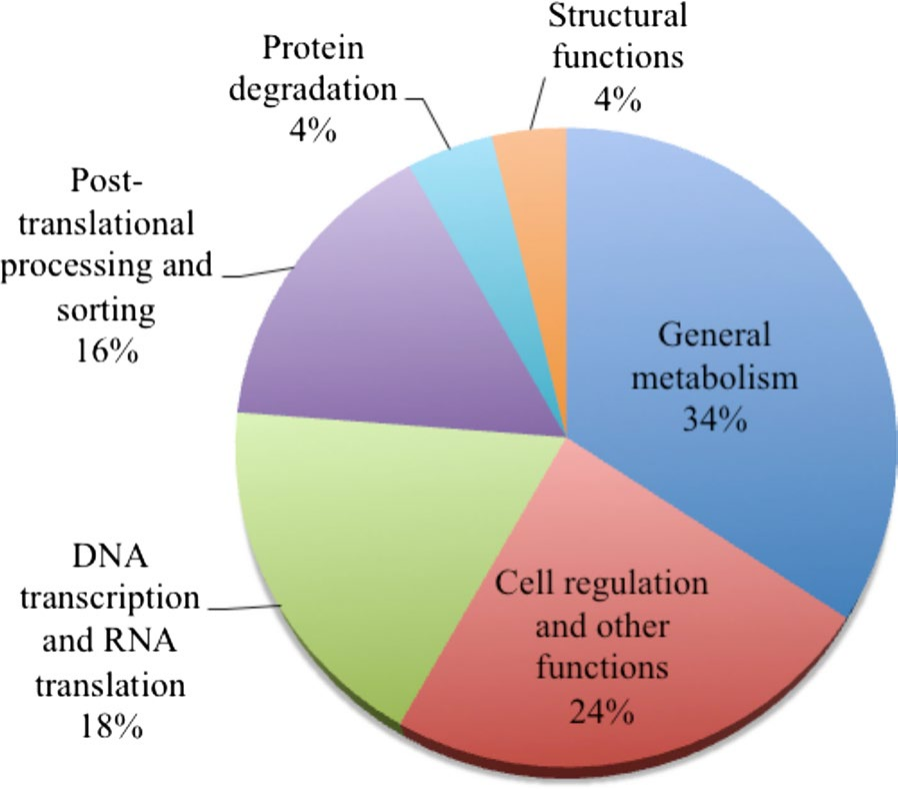
The putative cellular protein transcripts (non-toxins) from *B. colombiensis* according to their cellular functions

**Fig. 2:**
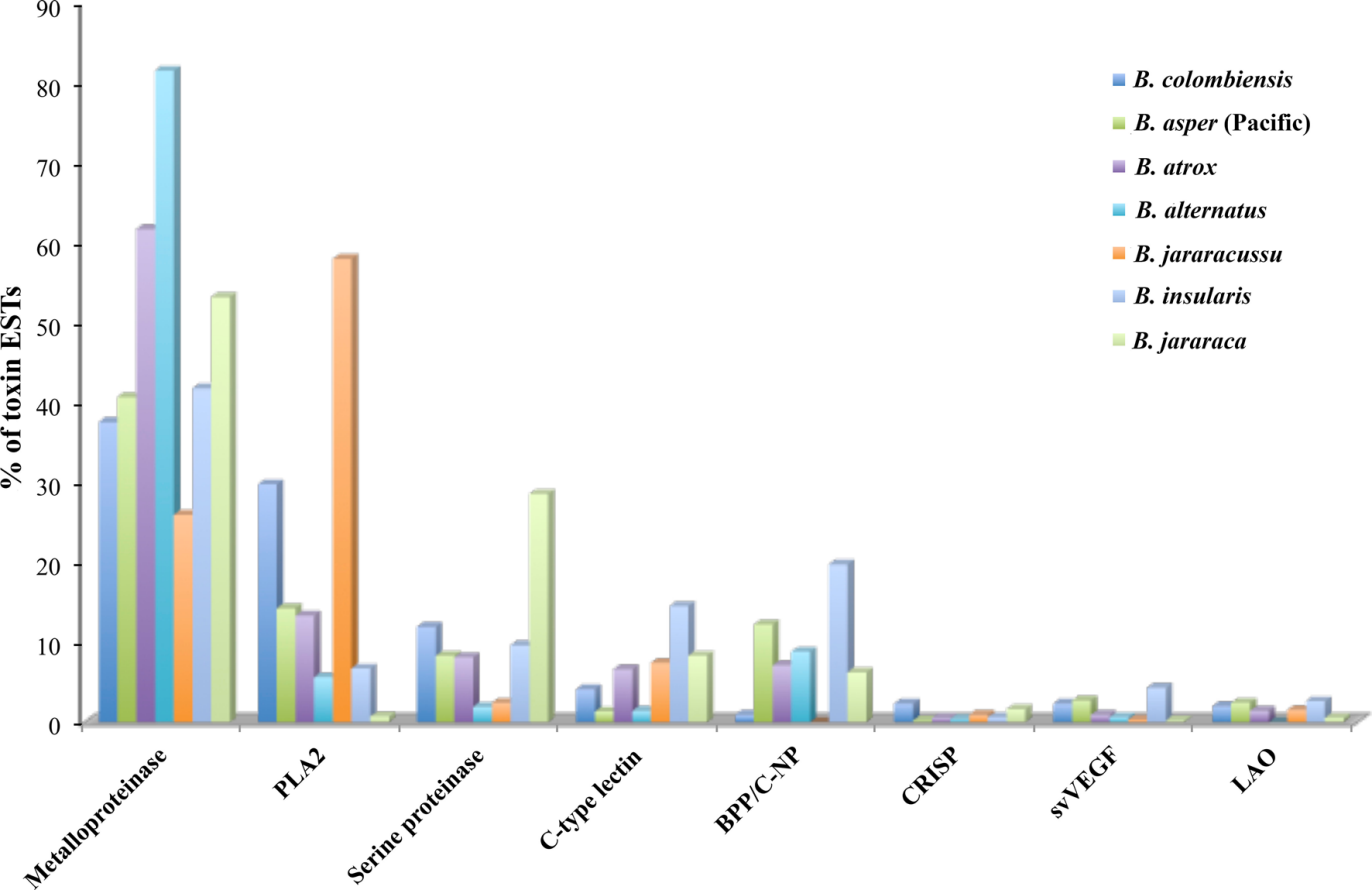
Relative abundance of the major toxin families in *Bothrops* venom gland transcriptomes. The abundance of transcripts is expressed as a percentage of the total toxin transcripts and was calculated by dividing the number of ESTs of each toxin family by the total number of toxin ESTs reported in each study. The data sources other than *B. colombiensis* were as follows: *B. asper* (Pacific) [65], *B. atrox* [25], *B. alternatus* [24], *B. jararacussu* [64], *B. insularis* [27], and *B. jararaca* [27]. The percentage of each toxin transcript of individual *Bothrops* species is shown in the Additional file 3
