# Supplementary material for: Gene expression profiling of the venomgland from the Venezuelan mapanare (Bothrops colombiensis) using expressed sequence tags (ESTs)
Source: BMC Mol Biol. 2016 Mar 5;17:7. doi: 10.1186/s12867-016-0059-7 (PMC4779267; doi:10.1186/s12867-016-0059-7)
Supplement: Supplementary file 1 — 10.1186/s12867-016-0059-7 Summary of statistics after clustering and assembly of 729 EST sequences. [file 12867_2016_59_MOESM1_ESM.docx]

**Additional file 1 Summary of statistics after clustering and assembly of 729 EST sequences.**

|  | **No. of clusters** | **No. of ESTs** | **Redundancy (clones/clusters)** | **% of total** | **% of matching** |
| --- | --- | --- | --- | --- | --- |
| Toxins | 32 | 344 | 10.8 | 47.2 | 68.3 |
| No. of consensus sequences  (> 1 member) | 18 | 331 | 18.3 | 45.3 |  |
| No. of singletons | 13 | 13 | 1.0 | 1.9 |  |
| Non-toxins | 119 | 160 | 1.3 | 22.0 | 31.8 |
| No. of consensus sequences | 14 | 55 | 3.9 | 7.5 |  |
| No. of singletons | 105 | 105 | 1.0 | 14.4 |  |
| Unknown proteins (no functional attributes) | 64 | 87 | 1.4 | 11.9 | - |
| No. of consensus sequences | 12 | 35 | 2.9 | 4.8 |  |
| No. of singletons | 52 | 52 | 1.0 | 7.1 |  |
| No database match | 73 | 138 | 1.9 | 18.9 | - |
| No. of consensus sequences | 13 | 78 | 6.0 | 10.7 |  |
| No. of singletons | 60 | 60 | 1.0 | 8.2 |  |
| **Total** | **288** | **729** |  | **100** |  |
