# Supplementary material for: Gene expression profiling of the venomgland from the Venezuelan mapanare (Bothrops colombiensis) using expressed sequence tags (ESTs)
Source: BMC Mol Biol. 2016 Mar 5;17:7. doi: 10.1186/s12867-016-0059-7 (PMC4779267; doi:10.1186/s12867-016-0059-7)
Supplement: Supplementary file 2 — 10.1186/s12867-016-0059-7 A representative list of putative protein identity matches for expressed sequence tags (ESTs) obtained from randomly sequenced clones from B. colombiensis venom gland. [file 12867_2016_59_MOESM2_ESM.docx]

**Additional file 2** **A representative list of putative protein identity matches for expressed sequence tags (ESTs) obtained from randomly sequenced clones from *B. colombiensis* venom gland.**

| **dbEST no.** | **HIT ID** | **Putative identification** | **E value** | **%Identity** |
| --- | --- | --- | --- | --- |
| JZ880164 | P83512.2 | Snake venom metalloproteinase BaP1 [*Bothrops asper*] | 0.00E+00 | 85.8% |
| JZ880059 | ADO21503.1 | MP_III3 SVMP precursor, partial [*Bothrops neuwiedi*] | 5.20E-52 | 90.7% |
| JZ880060 | AAP78953.1 | Metalloprotease BOJUMET III, partial [*Bothrops jararacussu*] | 1.20E-36 | 95.5% |
| JZ880061 | Q7T1T4.1 | Snake venom metalloproteinase BjussuMP-2 [*Bothrops jararacussu*] | 6.50E-131 | 87.5% |
| JZ880062 | Q7T1T4.1 | Snake venom metalloproteinase BjussuMP-2 [*Bothrops jararacussu*] | 3.90E-76 | 86.4% |
| JZ880063 | Q7T1T4.1 | Snake venom metalloproteinase BjussuMP-2 [*Bothrops jararacussu*] | 1.80E-83 | 87.1% |
| JZ880165 | Q7T1T4.1 | Snake venom metalloproteinase BjussuMP-2 [*Bothrops jararacussu*] | 7.80E-103 | 88.5% |
| JZ880064 | Q7T1T4.1 | Snake venom metalloproteinase BjussuMP-2 [*Bothrops jararacussu*] | 3.70E-84 | 87.1% |
| JZ880065 | Q7T1T4.1 | Snake venom metalloproteinase BjussuMP-2 [*Bothrops jararacussu*] | 2.80E-110 | 87.6% |
| JZ880066 | BAP39904.1 | Metalloprotease P-II [*Protobothrops elegans*] | 1.60E-163 | 82.7% |
| JZ880067 | P83512.2 | Snake venom metalloproteinase BaP1 [*Bothrops asper*] | 0.00E+00 | 86.6% |
| JZ880068 | P83512.2 | Snake venom metalloproteinase BaP1 [*Bothrops asper*] | 0.00E+00 | 88.0% |
| JZ880069 | Q5XUW8.1 | Zinc metalloproteinase/disintegrin [*Bothrops insularis*] | 4.80E-179 | 88.7% |
| JZ880070 | Q5XUW8.1 | Zinc metalloproteinase/disintegrin [*Bothrops insularis*] | 8.30E-180 | 87.4% |
| JZ880071 | P83512.2 | Snake venom metalloproteinase BaP1 [*Bothrops asper*] | 0.00E+00 | 86.9% |
| JZ880072 | P83512.2 | Snake venom metalloproteinase BaP1 [*Bothrops asper*] | 0.00E+00 | 87.7% |
| JZ880073 | P83512.2 | Snake venom metalloproteinase BaP1 [*Bothrops asper*] | 0.00E+00 | 87.4% |
| JZ880074 | P83512.2 | Snake venom metalloproteinase BaP1 [*Bothrops asper*] | 0.00E+00 | 87.5% |
| JZ880075 | Q072L5.1 | Zinc metalloproteinase/disintegrin [*Bothrops asper*] | 0.00E+00 | 84.8% |
| JZ880076 | P83512.2 | Snake venom metalloproteinase BaP1 [*Bothrops asper*] | 0.00E+00 | 69.8% |
| JZ880077 | P83512.2 | Snake venom metalloproteinase BaP1 [*Bothrops asper*] | 0.00E+00 | 87.8% |
| JZ880078 | P83512.2 | Snake venom metalloproteinase BaP1 [*Bothrops asper*] | 0.00E+00 | 87.7% |
| JZ880079 | P83512.2 | Snake venom metalloproteinase BaP1 [*Bothrops asper*] | 0.00E+00 | 85.6% |
| JZ880080 | P83512.2 | Snake venom metalloproteinase BaP1 [*Bothrops asper*] | 1.40E-104 | 92.1% |
| JZ880081 | P83512.2 | Snake venom metalloproteinase BaP1 [*Bothrops asper*] | 1.40E-104 | 92.1% |
| JZ880082 | Q7T1T4.1 | Snake venom metalloproteinase BjussuMP-2 [*Bothrops jararacussu*] | 9.50E-63 | 87.3% |
| JZ880083 | Q7T1T4.1 | Snake venom metalloproteinase BjussuMP-2 [*Bothrops jararacussu*] | 9.40E-61 | 88.7% |
| JZ880084 | P83512.2 | Snake venom metalloproteinase BaP1 [*Bothrops asper*] | 1.60E-180 | 88.0% |
| JZ880085 | Q7T1T4.1 | Snake venom metalloproteinase BjussuMP-2 [*Bothrops jararacussu*] | 9.50E-155 | 88.7% |
| JZ880086 | P83512.2 | Snake venom metalloproteinase BaP1 [*Bothrops asper*] | 2.90E-133 | 93.5% |
| JZ880087 | BAP39904.1 | Metalloprotease P-II [*Protobothrops elegans*] | 1.50E-158 | 88.3% |
| JZ880088 | BAP39904.1 | Metalloprotease P-II [*Protobothrops elegans*] | 2.20E-152 | 85.1% |
| JZ880089 | Q98UF9.3 | Zinc metalloproteinase-disintegrin-like HF3 [*Bothrops jararacussu*] | 6.50E-162 | 92.7% |
| JZ880090 | AEJ31987.1 | Zinc metalloproteinase/disintegrin VMP-II [*Crotalus viridis viridis*] | 7.40E-42 | 94.6% |
| JZ880091 | ADO21503.1 | MP_III3 SVMP precursor, partial [*Bothrops neuwiedi*] | 2.50E-138 | 89.4% |
| JZ880092 | C9E1S0.1 | Zinc metalloproteinase-disintegrin-like VMP-III [*Agkistrodon piscivorus leucostoma*] | 9.90E-15 | 80.9% |
| JZ880093 | P86092.1 | Zinc metalloproteinase leucurolysin-B [*Bothrops leucurus*] | 1.40E-115 | 88.7% |
| JZ880094 | Q8AWI5.1 | Zinc metalloproteinase-disintegrin-like halysase [*Gloydius halys*] | 1.30E-125 | 88.2% |
| JZ880095 | AEJ31986.1 | Metalloproteinase 3 [*Crotalus adamanteus*] | 1.40E-98 | 89.4% |
| JZ880096 | P83512.2 | Snake venom metalloproteinase BaP1 [*Bothrops asper*] | 3.80E-82 | 81.1% |
| JZ880097 | AEJ31991.1 | Metalloproteinase 8 [*Crotalus adamanteus*] | 2.00E-171 | 65.0% |
| JZ880098 | Q8JIR2.1 | Zinc metalloproteinase/disintegrin-like HR1a [*Protobothrops flavoviridis*] | 2.80E-175 | 68.1% |
| JZ880099 | AAP78953.1 | Metalloprotease BOJUMET III, partial [*Bothrops jararacussu*] | 8.70E-28 | 91.2% |
| JZ880100 | P0C6B6.1 | Zinc metalloproteinase homolog-disintegrin albolatin [*Trimeresurus albolabris*] | 3.90E-175 | 72.9% |
| JZ880101 | P24605.3 | Basic phospholipase A_2_ homolog 2 [*Bothrops asper*] | 6.10E-93 | 100.0% |
| JZ880102 | G3DT18.1 | Acidic phospholipase A_2_ BmooPLA_2_ [*Bothrops moojeni*] | 8.10E-88 | 89.9% |
| JZ880103 | AFJ79208.1 | Acidic secretory phospholipase A_2_ sPLA_2_-II [*Bothrops diporus*] | 2.30E-84 | 88.4% |
| JZ880104 | G3DT18.1 | Acidic phospholipase A_2_ BmooPLA_2_ [*Bothrops moojeni*] | 3.20E-82 | 89.2% |
| JZ880105 | G3DT18.1 | Acidic phospholipase A_2_ BmooPLA_2_ [*Bothrops moojeni*] | 8.10E-88 | 89.9% |
| JZ880106 | P20474.2 | Basic phospholipase A_2_ myotoxin III [*Bothrops asper*] | 1.20E-74 | 96.5% |
| JZ880107 | AFJ79208.1 | Acidic secretory phospholipase A_2_ sPLA_2_-II [*Bothrops diporus*] | 1.30E-70 | 79.3% |
| JZ880108 | P04971.1 | Thrombin-like enzyme batroxobin [*Bothrops atrox*] | 2.90E-104 | 88.4% |
| JZ880109 | Q8QG86.1 | Snake venom serine protease BITS01A [*Bothrops insularis*] | 5.90E-179 | 94.2% |
| JZ880110 | P04971.1 | Thrombin-like enzyme batroxobin [*Bothrops atrox*] | 2.90E-100 | 99.3% |
| JZ880111 | P04971.1 | Thrombin-like enzyme batroxobin [*Bothrops atrox*] | 0.00E+00 | 96.5% |
| JZ880112 | Q8QG86.1 | Snake venom serine protease BITS01A [*Bothrops insularis*] | 1.50E-179 | 94.6% |
| JZ880113 | P04971.1 | Thrombin-like enzyme batroxobin [*Bothrops atrox*] | 7.70E-167 | 95.8% |
| JZ880114 | P04971.1 | Thrombin-like enzyme batroxobin [*Bothrops atrox*] | 0.00E+00 | 95.7% |
| JZ880115 | P04971.1 | Thrombin-like enzyme batroxobin [*Bothrops atrox*] | 0.00E+00 | 96.5% |
| JZ880116 | P04971.1 | Thrombin-like enzyme batroxobin [*Bothrops atrox*] | 0.00E+00 | 96.1% |
| JZ880117 | Q8QG86.1 | Snake venom serine protease BITS01A [*Bothrops insularis*] | 1.00E-177 | 93.8% |
| JZ880118 | Q8QG86.1 | Snake venom serine protease BITS01A [*Bothrops insularis*] | 1.50E-179 | 94.6% |
| JZ880119 | Q8QG86.1 | Snake venom serine protease BITS01A [*Bothrops insularis*] | 3.30E-178 | 94.2% |
| JZ880120 | Q8QG86.1 | Snake venom serine protease BITS01A [*Bothrops insularis*] | 8.20E-179 | 94.5% |
| JZ880121 | Q5W959.1 | Snake venom serine protease HS114 [*Bothrops jararaca*] | 0.00E+00 | 98.1% |
| JZ880122 | ABG26974.1 | Serine proteinase isoform 8 [*Sistrurus catenatus edwardsi*] | 3.60E-167 | 91.9% |
| JZ880123 | ETE60122.1 | Dolichyl-diphosphooligosaccharide--protein glycosyltransferase 48 kDa subunit, partial | 7.70E-71 | 97.3% |
| JZ880124 | AEU60002.1 | Parvalbumin [*Crotalus oreganus helleri*] | 2.00E-67 | 97.0% |
| JZ880166 | AEU60002.1 | Parvalbumin [*Crotalus oreganus helleri*] | 4.70E-64 | 94.5% |
| JZ880125 | AAR19276.1 | Venom gland cyclophilin, partial [*Bitis gabonica*] | 1.60E-104 | 96.8% |
| JZ880126 | BAN89446.1 | Waprin, partial [*Ovophis okinavensis*] | 3.20E-18 | 74.0% |
| JZ880127 | A7X4J4.1 | Waprin-Rha1 [*Rhabdophis tigrinus tigrinus*] | 4.00E-19 | 66.7% |
| JZ880128 | BAP39964.1 | C-type lectin F IX/X B [*Protobothrops flavoviridis*] | 5.00E-68 | 78.0% |
| JZ880129 | XP_007421024.1 | Protein Asterix [*Python bivittatus*] | 3.00E-69 | 98.0% |
| JZ880130 | ETE73855.1 | V-type proton ATPase subunit e 1 [*Ophiophagus hannah*] | 9.20E-47 | 96.3% |
| JZ880131 | ETE71213.1 | Elongation factor 1-alpha 1 [*Ophiophagus hannah*] | 8.00E-58 | 98.9% |
| JZ880132 | XP_007441421.1 | Protein transport protein Sec61 subunit beta [*Python bivittatus*] | 2.60E-60 | 99.0% |
| JZ880133 | ETE69723.1 | Selenoprotein [15 kDa], partial [*Ophiophagus hannah*] | 9.90E-49 | 95.2% |
| JZ880134 | XP_007438236.1 | Translocating chain-associated membrane protein 1 [*Python bivittatus*] | 2.10E-22 | 78.9% |
| JZ880135 | ETE71092.1 | Translocating chain-associated membrane protein 1, partial [*Ophiophagus hannah*] | 1.30E-155 | 97.7% |
| JZ880136 | BAP39957.1 | Cysteine-rich venom protein, partial [*Protobothrops flavoviridis*] | 3.10E-161 | 83.7% |
| JZ880137 | Q8JI40.1 | Cysteine-rich venom protein ablomin [*Gloydius blomhoffii*] | 3.50E-94 | 86.4% |
| JZ880138 | ETE59024.1 | Cysteine-rich protein 1 [*Ophiophagus hannah*] | 6.30E-49 | 98.7% |
| JZ880139 | Q90X24.1 | Snake venom vascular endothelial growth factor toxin [*Bothrops insularis*] | 3.00E-97 | 95.2% |
| JZ880140 | Q90X24.1 | Snake venom vascular endothelial growth factor toxin [*Bothrops insularis*] | 3.50E-96 | 94.5% |
| JZ880141 | Q90X24.1 | Snake venom vascular endothelial growth factor toxin [*Bothrops insularis*] | 3.00E-97 | 95.2% |
| JZ880142 | B5AR80.1 | L-amino-acid oxidase [*Bothrops pauloensis*] | 0.00E+00 | 95.0% |
| JZ880143 | Q6TGQ9.1 | L-amino-acid oxidase [*Bothrops jararacussu*] | 0.00E+00 | 97.7% |
| JZ880144 | X2JCV5.1 | L-amino acid oxidase [*Cerastes cerastes*] | 5.00E-39 | 96.0% |
| JZ880145 | BAP39952.1 | Bradykinin-potentiating and C-type natriuretic peptides, partial [*Protobothrops flavoviridis*] | 1.00E-11 | 86.0% |
| JZ880146 | XP_007444663.1 | GPI-linked NAD[P][+]--arginine ADP-ribosyltransferase 1-like, partial [*Python bivittatus*] | 5.00E-83 | 54.0% |
| JZ880147 | BAN82026.1 | Phospholipase B [*Protobothrops flavoviridis*] | 1.00E-140 | 93.0% |
| JZ880148 | F8S101.1 | Phospholipase B [*Crotalus adamanteus*] | 3.50E-107 | 96.9% |
| JZ880149 | XP_007425204.1 | Peroxiredoxin-6 [*Python bivittatus*] | 4.60E-149 | 92.8% |
| JZ880150 | XP_007425204.1 | Peroxiredoxin-6 [*Python bivittatus*] | 5.00E-149 | 93.0% |
| JZ880151 | XP_007421287.1 | Thioredoxin-like protein 1 [*Python bivittatus*] | 2.00E-150 | 94.0% |
| JZ880152 | ETE65237.1 | LysM and putative peptidoglycan-binding domain-containing protein 3 [*Ophiophagus hannah*] | 5.10E-55 | 94.6% |
| JZ880153 | XP_007427442.1 | L-lactate dehydrogenase B chain [*Python bivittatus*] | 1.00E-104 | 92.0% |
| JZ880154 | XP_007432529.1 | Zinc finger protein 414-like [*Python bivittatus*] | 2.00E-60 | 98.0% |
| JZ880155 | XP_007441781.1 | Adenylate cyclase type 8-like, partial [*Python bivittatus*] | 1.80E-144 | 95.2% |
| JZ880156 | BAN89425.1 | Phosphodiesterase [*Ovophis okinavensis*] | 0.00E+00 | 81.0% |
| JZ880157 | ETE67407.1 | Parathymosin, partial [*Ophiophagus hannah*] | 1.50E-75 | 94.7% |
| JZ880158 | XP_007429057.1 | Cathepsin B isoform X1 [*Python bivittatus*] | 6.60E-169 | 89.6% |
| JZ880159 | XP_007421695.1 | Syntenin-1 [*Python bivittatus*] | 4.00E-177 | 90.0% |
| JZ880160 | XP_007429591.1 | Calnexin isoform X1 [*Python bivittatus*] | 1.60E-156 | 89.1% |
| JZ880161 | XP_007421850.1 | Serine/arginine-rich splicing factor 12 isoform X1 [*Python bivittatus*] | 5.00E-55 | 95.2% |
| JZ880162 | XP_007435588.1 | Ferritin heavy chain A-like [*Python bivittatus*] | 3.60E-26 | 96.0% |
| JZ880163 | XP_007434504.1 | Adenylate kinase isoenzyme 5 isoform X1 [*Python bivittatus*] | 9.00E-120 | 90.3% |
