# Supplementary material for: Gene expression profiling of the venomgland from the Venezuelan mapanare (Bothrops colombiensis) using expressed sequence tags (ESTs)
Source: BMC Mol Biol. 2016 Mar 5;17:7. doi: 10.1186/s12867-016-0059-7 (PMC4779267; doi:10.1186/s12867-016-0059-7)
Supplement: Supplementary file 3 — 10.1186/s12867-016-0059-7 Relative contributions of the different putative toxins hits in each of the Bothrops snake venom gland transcriptome. [file 12867_2016_59_MOESM3_ESM.docx]

**Additional file 3** **Relative contributions of the different putative toxins hits in each of the *Bothrops* snake venom gland transcriptome.**

|  | % of total toxin transcripts | | | | | | | | | | |
| --- | --- | --- | --- | --- | --- | --- | --- | --- | --- | --- | --- |
|  | | *B. colombiensis* (this study) | *B. asper* (Car) [65] | *B. asper* (Pac) [65] | *B. atrox* [25] | *B. alternatus* [24] | *B. jararacussu* [64] | *B. insularis* [19, 27] | *B. insularis* [78] | *B. jararaca* [27] | *B. jararaca* (adult) [26] |
|  |  |  |  |  |  |  |  |  |  |  |  |
| Metalloproteinase | | 37.5 | 41.6 | 40.6 | 61.6 | 81.4 | 25.9 | 41.7 | 43.2 | 53.1 | 29.9 |
| PLA_2_ | | 29.7 | 17.8 | 14.2 | 13.3 | 5.6 | 57.9 | 6.7 | 5.4 | 0.7 | 9.5 |
| Serine proteinase | | 11.9 | 13.4 | 8.3 | 8.1 | 1.9 | 2.4 | 9.6 | 11.2 | 28.5 | 8.1 |
| WAP | | 5.5 | 0.09 | 0 | nd | nd | nd | nd | nd | nd | nd |
| C-type lectin | | 4.1 | 1 | 1.3 | 6.6 | 1.4 | 7.4 | 14.5 | 14.2 | 8.3 | 22.3 |
| Nucleotidases | | 3.2 | 0.9 | 0.4 | nd | 0.6^b^ | nd | nd | nd | nd | 0.3 |
| CRISP | | 2.3 | 0.8 | 0.2 | 0.5 | 0.4 | 0.9 | 0.6 | 1.5 | 1.6 | 1.0 |
| svVEGF | | 2.3 | 0.9 | 2.7 | 0.9 | 0.6 | nd | 4.3 | 4.7 | 0.2 | 1.5 |
| LAO | | 2 | 4.2 | 2.4 | 1.4 | 0 | 1.5 | 2.6 | 3.5 | 0.5 | 2.4 |
| BPP/C-NP | | 0.9 | 15 | 12.2 | 7.1 | 8.8 | nd | 19.7 | 15.8 | 6.2 | 23.2 |
| Phospholipase B | | 0.6 | nd | nd | 0.7^b^ | nd | nd | nd | nd | nd | nd |
| Others | | - | 4.1^a^ | 17.5^a^ | 0.5^c^ | 0.7^d^ | 3.8^e^ | 0.3^f^ | 0.4^f^ | 0.9^c^ | 1.5^g^ |

Putative toxin names are abbreviated as in Table 2; nd = not determined

The cited references are indicated in parentheses.

^a^Others include glutaminyl cyclase, crobra venom factor, crotamine, sarafotoxin, kunitz-type inhibitor, kazal-type inhibitor, hyaluronidase, ohanin, and three-finger toxin.

^b^Transcript was not classified as toxin.

^c^PLA_2_ inhibitor.

^d^Others include taicatoxin-like protein, dipeptidylpeptidase IV, prothrombin activator, muscarinic-like toxin, neurptoxin 6, and castrocollastatin precursor.

^e^Vascular apoptosis inducing protein and ablomin like protein.

^f^Nerve growth factor.

^g^Others include nerve growth factor, ohanin precursor, and hyaluronidase.
